# A Stable Bioisostere of Ester‐Linked Ubiquitin Chains Enables Decoding of Protein Interactors

**DOI:** 10.1002/cbic.202500749

**Published:** 2025-12-17

**Authors:** Yoshinori Taguchi, Takuya Tomita, Takuma Nishizawa, Dai Nakamura, Showmitra Saha, Takanori Oyoshi, Kohei Sato, Nobuyuki Mase, Yasushi Saeki, Tetsuo Narumi

**Affiliations:** ^1^ Graduate School of Medical Photonics Shizuoka University 3‐5‐1 Jouhoku, Chuo‐ku Hamamatsu 432–8561 Shizuoka Japan; ^2^ Division of Protein Metabolism Institute of Medical Science The University of Tokyo 4‐6‐1 Shirokanedai Minato‐ku 108–8639 Tokyo Japan; ^3^ Graduate School of Integrated Science and Technology Department of Engineering Shizuoka University 3‐5‐1 Jouhoku, Chuo‐ku Hamamatsu 432–8561 Shizuoka Japan; ^4^ Protein Metabolism Project Tokyo Metropolitan Institute of Medical Science 2‐1‐6 Kamikitazawa Setagaya‐ku 156–8506 Tokyo Japan; ^5^ Graduate School of Integrated Science and Technology Department of Science Shizuoka University 836 Ohya Shizuoka 422–8529 Suruga Japan; ^6^ Research Institute of Green Science and Technology Shizuoka University 3‐5‐1 Jouhoku, Chuo‐ku Shizuoka 432–8561 Hamamatsu Japan; ^7^ Faculty of Engineering Department of Applied Chemistry and Biochemical Engineering Shizuoka University 3‐5‐1 Jouhoku, Chuo‐ku Shizuoka 432–8561 Hamamatsu Japan; ^8^ Graduate School of Science and Technology Shizuoka University 3‐5‐1 Jouhoku, Chuo‐ku Shizuoka 432–8561 Hamamatsu Japan

**Keywords:** ester‐linked ubiquitin chain, ester‐to‐amide replacement, interactome analysis, protein chemical synthesis

## Abstract

Protein ubiquitination is a pivotal posttranslational modification that regulates diverse biological processes depending on the type of ubiquitin chain linkage. Recently, ester‐linked ubiquitin chains have been identified, yet their inherent hydrolytic instability has posed a significant challenge for biochemical investigations. In this study, a stable and isosteric amide analog of an ester‐linked ubiquitin dimer, is chemically synthesized in which serine (Ser) at position 20 of the proximal ubiquitin is replaced with 2,3‐diaminopropionic acid (Dap). The desired amide analog is synthesized using a convergent approach involving the sequential chemoselective ligation of three peptide fragments generated through Fmoc‐based solid‐phase peptide synthesis. Employing this chemically robust ubiquitin probe, a previously unrecognized interaction is uncovered between Ser20‐linked ubiquitin chains and spliceosome‐associated factors, notably ubiquitin‐specific protease 39. These findings highlight the potential of the ester‐to‐amide bioisosteric strategy to unlock mechanistic insights into atypical ubiquitin modifications. The approach not only circumvents the intrinsic instability of ester‐linked ubiquitin chains but also provides a broadly applicable framework for dissecting their biological roles, paving the way for future discoveries in ubiquitin signaling.

## Graphical abstract

An ester‐to‐amide analog of an ester‐linked ubiquitin dimer is chemically synthesized through a convergent approach involving the sequential chemoselective ligation of three peptide fragments generated through Fmoc‐based solid‐phase peptide synthesis. The hydrolysis‐resistant ubiquitin dimer served as a chemical probe in the identification of potential reader of the ester‐linked ubiquitin chain.

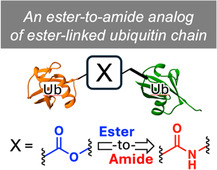

## 1 Introduction

**Figure 1:**
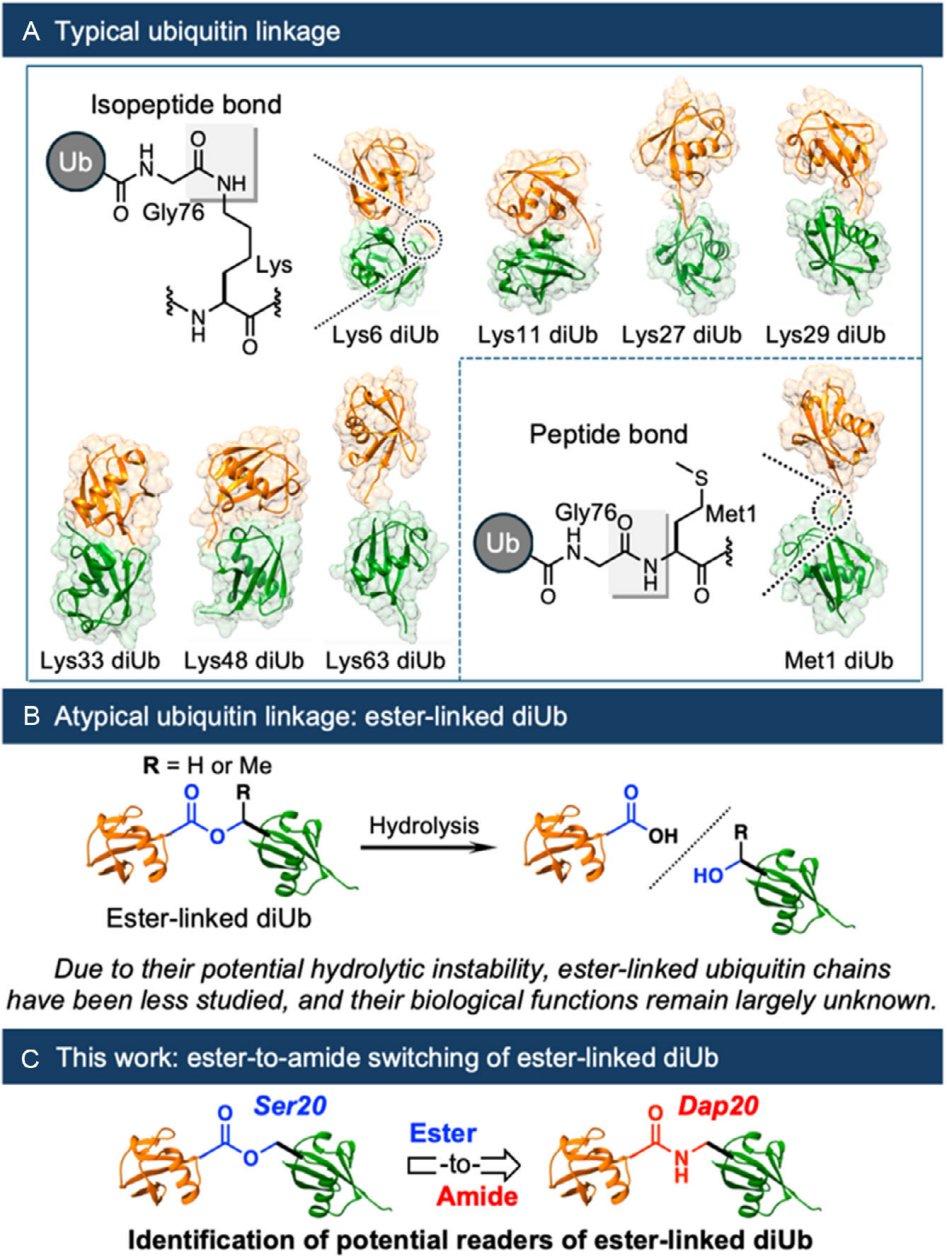
A) Ubiquitin linkage types and their contribution to the topological diversity of ubiquitin chains. (Protein data bank IDs: diubiquitin (diUb): Lys6, 2XK5; Lys11, 2XEW; Lys27, 6QML; Lys29, 4S22; Lys33, 4XYZ; Lys48, 5GOI; Lys63, 2JF5; and Met1, 2W9N) B) Characteristics of ester‐linked ubiquitin as an atypical ubiquitin linkage. C) Ester‐to‐amide substitution strategy for ester‐linked diUb to facilitate the identification of its readers.

Ubiquitin is a small globular protein used in various cellular processes for posttranslational modification. Ubiquitination is mediated by three classes of enzymes: E1 ubiquitin‐activating enzymes, E2 ubiquitin‐conjugating enzymes, and E3 ubiquitin ligases, which attach ubiquitin to substrate proteins or other ubiquitin molecules, leading to mono‐ or polyubiquitination.^[^ ^1^ ^]^ Ubiquitin chains are primarily formed through intermolecular isopeptide bonds between the *C*‐terminal carboxy group of glycine (Gly) 76 in the distal ubiquitin and *ε*‐amino group of its seven lysine (Lys) residues (Lys6, Lys11, Lys27, Lys29, Lys33, Lys48, and Lys63) or through peptide bonding with the α‐amino group of *N*‐terminal methionine (Met) in the proximal ubiquitin (**Figure** **1**A).^[^ ^2^, ^3^, ^4^ ^]^ These linkage differences provide topological diversity to ubiquitin chains, which is recognized by ubiquitin‐chain‐binding factors known as readers.

In addition to these canonical covalent linkages, emerging evidence indicates that ubiquitin chains are also formed through serine (Ser) and threonine (Thr) residues *via* thioester or oxyester bonding, respectively.^[^ ^4^ ^,^ ^5^ ^]^ In particular, E3 ligase HOIL‐1L, a component of the linear ubiquitin chain assembly complex, induces the Ser‐ or Thr‐linked ubiquitination of several physiological proteins and catalyzes ester bond formation between ubiquitin moieties at Thr12, Thr14, Ser20, Thr22, and Thr55, at least in vitro (Figure 1B).^[^ ^6^, ^7^, ^8^, ^9^ ^]^ These atypical linkages have been suggested to recruit negative regulators of Toll‐like receptor signaling and proteins that enhance inflammatory mediator production. However, the biological significance of these ester‐linked ubiquitin remains to be fully elucidated. The production of ubiquitin chains of high purity and homogeneous length by chemical protein synthesis is essential to further investigate the biological implications of these atypical ubiquitin chains.^[^ ^9^, ^10^, ^11^, ^12^, ^13^ ^]^

Previously, Brik and coworkers reported the total chemical synthesis of ester‐linked monoubiquitinated α‐globin and investigated its interaction with deubiquitinases.^[^ ^14^ ^]^ This earlier study established a novel platform for the synthesis of high‐quality ester‐linked ubiquitinated proteins.

Although a powerful method for the preparation of ester‐linked ubiquitinated proteins has been developed, physiological studies of ester‐linked ubiquitin chains remain scarce, mainly due to the lack of suitable chemical probes. Moreover, the intrinsic hydrolytic lability of the ester linkage suggests the potential susceptibility of such chains to degradation under physiological conditions. To address this limitation, the development of hydrolysis‐resistant analogs of ester‐linked ubiquitin chain is crucial.

This study presents a chemical synthesis route for an ester‐to‐amide analog of ester‐linked diubiquitin (diUb) for proteomic analysis. This ester‐to‐amide substitution strategy allows the preparation of chemically stable ubiquitin chain analogs that can serve as surrogates for inherently labile ester‐linked chains. Additionally, mass‐spectrometry‐based interactome analysis demonstrates that amide analogs, when used as chemical protein probes, exhibit binding affinity for unique readers.

## 2 Results and Discussion

### 2.1 Design and Synthesis of 2,3‐Diaminopropionic Acid (Dap)20‐ and Ser20‐Linked diUb

To address hydrolytic instability inherent to ester‐linked ubiquitin chains, we designed an amide analog of Ser20‐linked diUb (Figure 1C). This design holds potential to synthesize distinct higher‐order structures and enable specific interactions with readers, offering new possibilities beyond conventional isopeptide linkages.

**Figure 2:**
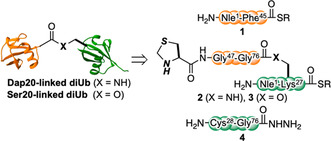
Synthetic strategy for Dap20‐ and Ser20‐linked diUb.

**Figure** **2** illustrates the overall strategy for synthesizing Dap20‐ and Ser20‐linked diUb. This strategy involves dividing the ubiquitin chain into three key peptide fragments. This modular strategy enables the selective preparation of either amide‐ or ester‐linked ubiquitin chains by interchanging the middle‐branched fragment with its corresponding analog. Peptide fragments (**1** and **4**) corresponding to the distal Ub(1–45) and proximal Ub(28–76) sequences of ubiquitin, are synthesized using standard 9‐fluorenylmethyloxycarbonyl (Fmoc)‐based solid‐phase peptide synthesis (SPPS) (see the Supporting Information).

**Scheme 1:**
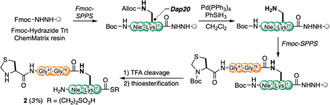
Synthetic scheme for the branched fragment via Dap20.

The synthesis process of the peptide fragment **2** is presented in **Scheme** **1**. The peptide fragment **2**, bearing the Dap side chain, was synthesized using Fmoc‐Dap‐OH, with the side chain protected with allyloxycarbonyl (Alloc) to enable the peptide elongation of the distal [Thz^47^–Gly^76^] sequence at position 20. Initially, following the elongation of the peptide chain corresponding to [Gln^2^–Lys^27^], including Dap(Alloc), *N*‐terminal Boc‐norleucine (Nle) was introduced as a Met surrogate to avoid Met oxidation.^[^ ^15^ ^]^ Following Alloc deprotection, the distal peptide chain was elongated to correspond to the [Gly^47^–Gly^76^] sequence, extending from the amino group of the Dap side chain. Boc‐thiazolidine‐4‐carboxylic acid (Thz), a Cys derivative, was coupled at the *N*‐terminus as a peptide ligation site.^[^ ^16^ ^,^ ^17^ ^]^ Finally, trifluoroacetic acid (TFA) cleavage and thioesterification with sodium 2‐mercaptoethanesulfonate (MESNa) were performed to obtain peptide thioester **2**.^[^ ^17^ ^]^ Simultaneously, peptide fragment **3** was synthesized *via* Fmoc‐SPPS (see the Supporting Information). Initially, a peptide corresponding to the [Asp^21^–Lys^27^] sequence of ubiquitin was elongated *via* Fmoc‐SPPS, and side‐chain‐unprotected Fmoc‐Ser‐OH was coupled at position 20. The *N*‐terminal Fmoc protecting group was then removed, and Fmoc‐Pro‐OH was coupled. Subsequently, Alloc‐protected Gly was coupled to the unprotected hydroxyl group of Ser using 4‐dimethylaminopyridine and *N*,*N*′‐diisopropylcarbodiimide to form an ester linkage. Next, the proximal [Gln^2^–Glu^18^] peptide was elongated, and Nle was introduced as a Met surrogate. The Alloc deprotection of Gly was performed *via* treatment with Pd(PPh_3_)_4_ and PhSiH_3_, followed by the elongation of the remaining [Gly^47^–Gly^75^] sequence and coupling of Boc‐Thz‐OH. Finally, after TFA cleavage, peptide hydrazide was converted to peptide thioester, yielding branched fragment **3**.

**Figure 3:**
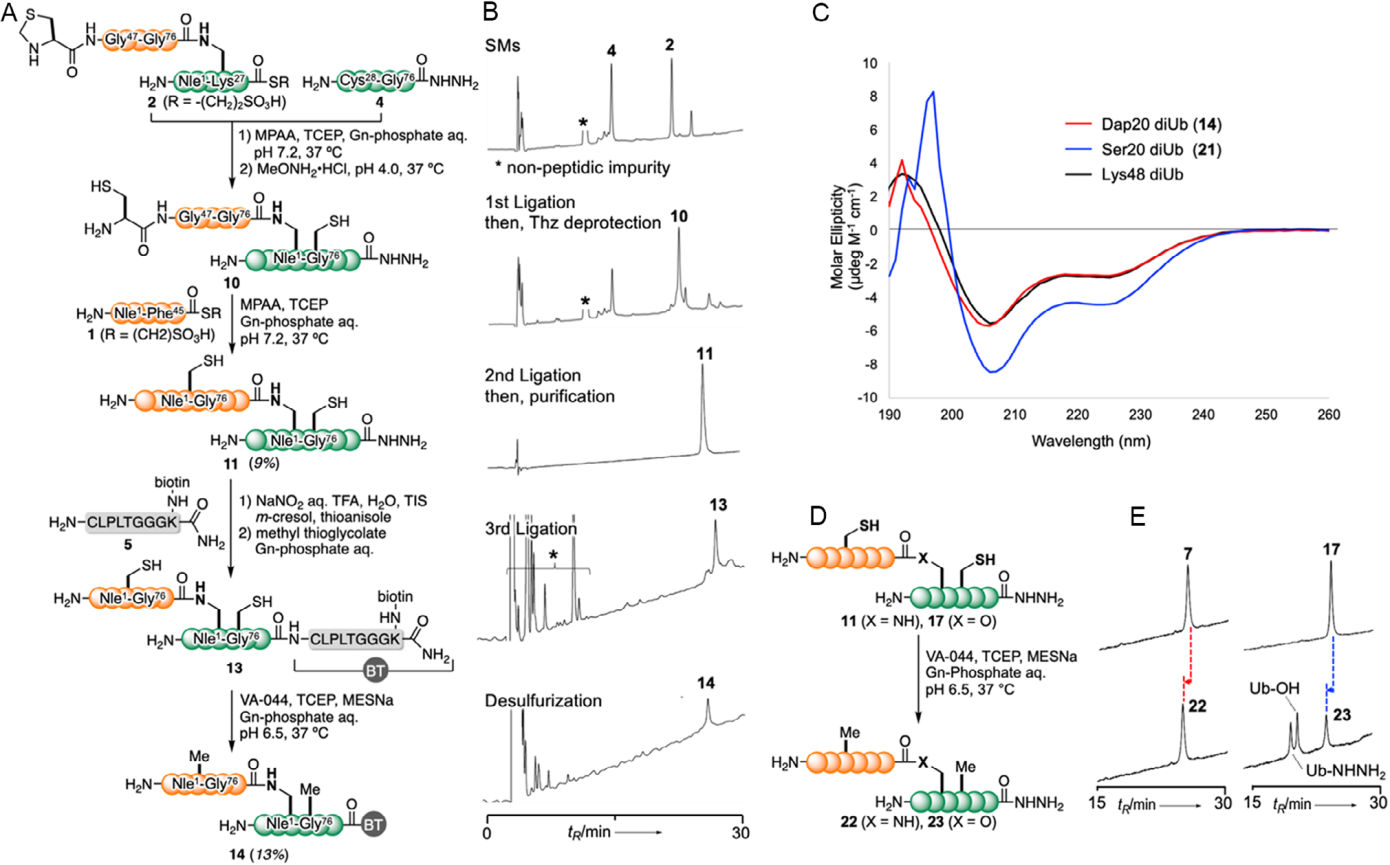
A) Synthesis of Dap20‐linked diUb **14**. B) HPLC profiles recorded during Dap20‐linked diUb synthesis. C) CD spectral analysis results for Dap20 diUb **14**, Ser20 diUb **21** and Lys48 diUb in 10 mM phosphate aq. pH 7.2. D) Chemical stability of Dap20‐linked and Ser20‐linked diUb in desulfurization condition and E) HPLC profile recorded during chemical stability accessment.

Having synthesized all peptide fragments, we prepared the Dap20‐linked diUb. The high‐performance liquid chromatography (HPLC) profiles recorded during each step are illustrated in **Figure** **3**B. Branched peptide **2** was ligated with cysteinyl peptide **4** under peptide ligation conditions (100 mM 4‐mercaptophenylacetic acid (MPAA), 50 mM tris(2‐carboxyethyl)phosphine hydrochloride (TCEP), 6 M guanidine hydrochloride (Gn), 200 mM phosphate, pH 7.2, 37 °C).^[^ ^18^ ^]^ The subsequent treatment of ligated peptide with methoxyamine (MeONH_2_) successfully removed the Thz protecting group to obtain **10**. The successive introduction of peptide thioester **1** under neutral conditions yielded the diUb precursor **11** through a one‐pot reaction.

To comprehensively explore the binding proteins of Dap20‐linked diUb, we introduced a biotin‐containing linker into Dap20‐linked diUb. Treating **11** with aqueous sodium nitrite, *m*‐cresol, thioanisole, triisopropylsilane, and water in TFA converted the *C*‐terminal acyl hydrazide to an acyl azide. This was followed by thioester formation and subsequent native chemical ligation with biotinylated peptide **5**, producing intermediate **13**, which was then treated with 2,2′‐azobis[2‐(2‐imidazolin‐2‐yl)propane] dihydrochloride (VA‐044), TCEP, and MESNa for desulfurization to yield biotinylated Dap20‐linked diUb **14** in 13%.^[^ ^19^ ^]^

The Ser20‐linked diUb **21** was synthesized through a similar process as Dap20‐linked diUb **14** (see the Supporting Information).

Additionally, similar to the circular dichroism (CD) spectrum of Lys48‐linked diUb, the CD spectra of Dap20‐ and Ser20‐linked diUb (**14** and **21**) exhibited characteristic negative peaks at 208 and 226 nm, indicating that they were folded correctly (Figure 3C).^[^ ^20^ ^]^

Next, we examined the chemical stability of Dap20‐ and Ser20‐linked diUb under desulfurization conditions. The Dap20‐linked diUb precursor **11** was treated with VA‐044, TCEP, and MESNa, to afford Dap20‐linked diUb **22** without any detectable cleavage of the peptide bond, as anticipated. In contrast, desulfurization of the Ser20‐linked diUb precursor **17** produced Ser20‐linked diUb **23** together with a ubiquitin monomer as a byproduct, which is attributable to the hydrolysis of the ester linkage under these conditions.

These findings suggested that replacing ester bonds with amide bonds was an effective strategy to prepare chemically stable ubiquitin chain analogs as surrogates for inherently labile ester‐linked chains.

### 2.2 Interactome Analysis Using the Dap20‐Linked diUb Probe

**Figure 4:**
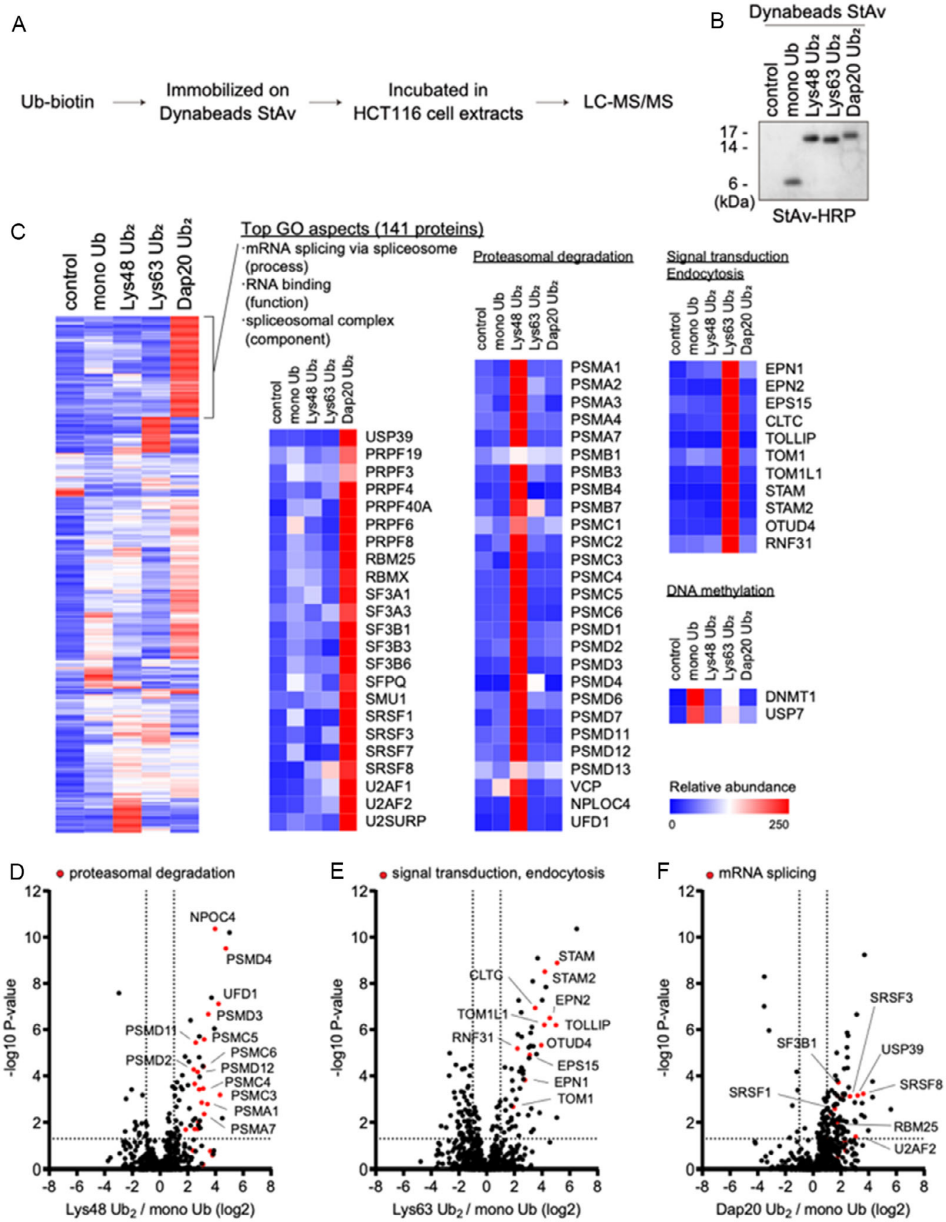
Mass‐spectrometry‐based interactome analysis of Dap20‐linked diUb. A) Experimental scheme for interactome analysis using biotinylated ubiquitin and HCT116 cell extracts. B) Boiled Dynabeads containing the indicated ubiquitin, with eluted biotinylated ubiquitins analyzed via Western blotting using horseradish‐peroxidase‐conjugated StAv. C) Heatmap of ubiquitin‐readers identified by LC‐MS/MS analysis (*n* = 3) with hierarchical clustering. The whole interactome is shown on the left, with clusters enriched in each ubiquitin chain displayed separately. The strongest interactors of Dap20‐linked diUb (141 proteins) were further subjected to a gene ontology (GO) analysis. The top GO term for each category (process, function, and component) is indicated. D–F) Volcano plots of the log2 ratios for the (D) Lys48 Ub2 versus mono Ub, (E) Lys63 Ub2 versus mono Ub, and (F) Dap20 Ub2 versus mono Ub and the unadjusted *p*‐values in one‐way ANOVA for individual proteins. Proteins related to (D) proteasomal degradation, (E) signal transduction and endocytosis, and (F) mRNA splicing that were picked up in the (C) were highlighted in red. Auxiliary lines are shown at a fold change of +2 or −2 on the *x*‐axis and a *p*‐value of 0.05 on the *y*‐axis. The whole list of identified proteins are presented in Table S1, Supporting Information.

To identify the readers of Dap20‐linked diUb, we enzymatically generated biotinylated Lys48‐ and Lys63‐linked ubiquitin chains. These chains were then separated based on the number of ubiquitin moieties using cation exchange chromatography (see the Supporting Information). Subsequently, biotinylated diUb species and monoubiquitin were immobilized on streptavidin (StAv)‐coated magnetic beads and subjected to an in vitro pulldown assay using HCT116 whole‐cell extracts (**Figure** **4**A,B).^[^ ^21^ ^]^

Coprecipitated proteins were detected by label‐free quantitative mass spectrometry, which identified 722 interactors in total, each displaying appreciable selectivity for specific ubiquitin linkages (Figure 4C, also see the Supporting Information). For instance, Lys48‐linked diUb primarily interacted with proteasome subunits and the unfoldase VCP, while Lys63‐linked diUb was associated with clusters of signal transduction‐ and endocytosis‐related molecules, consistent with previous findings.^[^ ^21^ ^]^ Monoubiquitin bound to the DNA methylation factors DNMT1 and the deubiquitinase USP7, with DNMT1 known to contain two binding sites for monoubiquitin.^[^ ^22^ ^]^ These results support that our interactome analysis identified a highly selective and functionally relevant set of ubiquitin‐binding proteins.

Unexpectedly, we found that Dap20‐linked diUb interacted predominantly with spliceosome factors, including pre‐mRNA‐processing‐splicing factors, RNA‐binding motif proteins, splicing factors, and serine‐ and arginine‐rich splicing factors, as indicated by the GO analysis. This finding is particularly intriguing, given that spliceosome dynamics are known to be regulated by the reversible ubiquitination of its subunits. For instance, PRPF3 and PRPF31 are ubiquitinated by the E3 ligase PRPF19, which stabilizes the U4/U6·U5 tri–snRNP complex by enhancing interactions between spliceosome components.^[^ ^23^ ^,^ ^24^ ^]^ However, the regulatory role of ubiquitination in spliceosome function remains incompletely understood, suggesting possible involvement of atypical ubiquitin chains.

### 2.3 Direct Binding of Dap20‐Linked diUb to USP39 In Vitro

Among identified spliceosome components, we found at least two known ubiquitin‐binding proteins, PRPF8 and USP39 (ubiquitin‐specific peptidase 39, also known as U4/U6·U5 tri‐snRNP‐associated protein 2). PRPF8 has been reported to directly recognize Lys63‐linked chains.^[^ ^23^ ^,^ ^24^ ^]^ In contrast, the binding preference of USP39 for ubiquitin linkage remained unclear, although its involvement in the regulation of Lys6‐ and Lys48‐linked chains was reported.^[^ ^25^ ^,^ ^26^ ^]^ This prompted us to investigate whether USP39 served as a direct binding partner to Dap20‐linked diUb.

**Figure 5:**
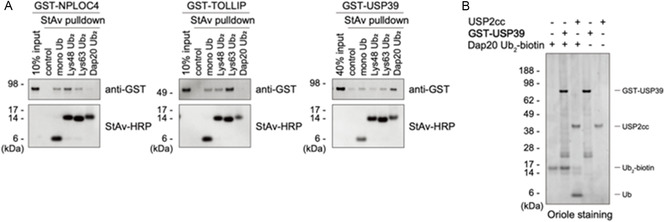
Interaction between Dap20‐linked diUb and reader. A) Biotinylated ubiquitins conjugated to magnetic beads, as shown in Figure 4B, incubated with indicated glutathione S‐transferase (GST)‐tagged proteins, followed by StAv pulldown. Coprecipitated proteins were analyzed via immunoblotting using an anti‐GST antibody and a horseradish‐peroxidase‐conjugated StAv. B) Sensitivity of Dap20‐linked diUb to the deubiquitinating enzyme USP2cc or GST‐USP39 analyzed utilizing SDS‐PAGE and Oriole staining.

To test this, we purified recombinant GST‐tagged USP39 as well as known readers of the Lys48‐ and Lys63‐linked chains, NPLOC4 and TOLLIP, respectively, for direct binding assay (see the Supporting Information).^[^ ^21^ ^]^ GST‐NPLOC4 bound most strongly to Lys48‐linked diUb, while GST‐TOLLIP bound to Lys63‐linked diUb (**Figure** **5**A). The pulldown assay further revealed that GST‐USP39 interacted most strongly with Dap20‐linked diUb, suggesting that USP39 might serve as its reader at least in vitro. Given that GST‐USP39 also exhibited slight binding affinity for other tested ubiquitins, we do not exclude the possibility that USP39 serves as a reader of Lys‐linked chains *in cellulo*.

USP39 contains a deubiquitinating domain; however, its enzymatic activity remains controversial. This uncertainty arises from the absence of conserved histidine and cysteine residues, which are essential for the deubiquitinating activity of USP proteins.^[^ ^27^ ^]^ Although several earlier reports describing USP39 as an active deubiquitinase,^[^ ^26^ ^,^ ^28^, ^29^, ^30^ ^]^ a recent study indicated that its deubiquitinating activity is fully inactive in vitro and in cellulo.^[^ ^31^ ^]^ Consequently, we tested the deubiquitinase activity of USP39 toward Dap20‐linked diUb. We found that GST‐USP39 did not cleave Dap20‐linked diUb, whereas the pan‐deubiquitinase USP2cc readily cleaved it, suggesting that USP39 did not deubiquitinate at least Dap20‐linked ubiquitin chains (Figure 5B). We speculate that, similar to PRPF8, USP39 functions in the spliceosome through ubiquitin recognition or other unidentified mechanisms.

Collectively, our novel chemical probe identified USP39 as a potential reader of Ser20‐linked ubiquitin chains. Given the limited knowledge regarding endogenous substrates and E3 ligases for Ser20‐linked ubiquitin and absence of reported Ser20‐linked chains in spliceosomes, the physiological relevance of our in vitro observations remains to be determined. Nevertheless, these findings were obtained solely using our novel chemical probe, providing a foundation for future studies. We anticipate that diverse functional bioisosteres will be developed to boost the investigation of ester‐linked ubiquitin chains.

## 3 Conclusion

As an analog of Ser20‐linked ubiquitin chains, we synthesized Dap20‐linked diUb, an amide analog wherein an easily hydrolysable ester bond was replaced with a more stable amide bond by chemical protein synthesis. Mass spectrometry‐based interactome analysis revealed that the interaction preference profile of Dap20‐linked diUb differed completely from those of typical Lys‐linked chains, suggesting that it might function as a unique ubiquitin code. In particular, USP39 was directly and strongly interacted with Dap20‐linked diUb, suggesting a potential role for USP39 in its decoding. The present study offers novel insights into the domain of ester‐linked ubiquitin research, a field previously hindered by its hydrolytic instability.

## Conflict of Interest

The authors declare no conflict of interest.

## Supporting information

Supplementary Material

## Data Availability

The data that support the findings of this study are openly available in PRIDE, reference number PXD069069.
