# Supplementary material for: A Stable Bioisostere of Ester‐Linked Ubiquitin Chains Enables Decoding of Protein Interactors
Source: Chembiochem. 2025 Dec 17;27(1):e202500749. doi: 10.1002/cbic.202500749 (PMC12789890; doi:10.1002/cbic.202500749)
Supplement: Supplementary file 1 — Supplementary Material [file CBIC-27-e202500749-s001.pdf]

Supporting Information

Table of contents

1. General information…..……………..…………………………………………...… S2
2. Experimental procedure for the synthesis of peptide fragments……………. S3
3. Experimental procedure of peptide ligation and desulfurization………..... S10
4. Experimental procedure of interactome analysis………..……………… S16

1. General information

Fmoc-Solid Phase Peptide Synthesis (SPPS). Fmoc SPPS was performed with a manual synthesis or an automated microwave-assisted synthesis. The microwave-assisted SPPS using a Biotage Initiator+ Alstra peptide synthesizer with a 10 mL open-type vial was conducted as follows: coupling with amino acids except for Cys(Trt) or Arg(Pbf): Fmoc-protected amino acid, ethyl 2-cyano-2-(hydroxyamino)acetate (Oxyma), and *N*,*N*-diisopropylcarbodiimide (DIPCI) (4.0 equivalents each) in *N*,*N*-dimethylformamide (DMF) (0.5 M), 5 min, 75 °C under microwave irradiation; coupling with Cys(Trt) or Arg(Pbf): Fmoc-protected amino acid, Oxyma and DIPCI (4.0 equivalents each) in DMF (0.5 M), 60 min, r.t.; Fmoc removal: 20% (v/v) piperidine in DMF, 3 min, 50 °C under microwave irradiation. The reaction temperatures were monitored by an IR sensor. Except where specifically noted, the manual synthesis was conducted as follows: 1. (coupling) Fmoc-protected amino acid, Oxyma, and DIPCI (4.0 equivalents each) in DMF (0.3 M), 60 min, r.t.; 2. (Fmoc removal) 20% (v/v) piperidine in DMF, 10 min, r.t.

Characterization data. High-resolution mass spectra were recorded on Bruker Daltonics compact (ESI-MS) spectrometers in the positive detection mode.

HPLC condition. For HPLC separations, a COSMOSIL 5C_{18}-AR-II analytical column (Nacalai Tesque, 4.6 × 250 mm, flow rate 1.0 mL min^{-1}), COSMOSIL 5C_{18}-AR-II preparative column (Nacalai Tesque, 20 × 250 mm, flow rate 10 mL min^{-1}), COSMOSIL 5C_8-AR300 analytical column (Nacalai Tesque, 4.6 × 250 mm, flow rate 1.0 mL min^{-1}), YMC-Triart C8 (YMC, 10 × 250 mm, flow rate 4.7 mL min^{-1}), COSMOSIL Protein-R (Nacalai Tesque, 4.6 × 250 mm, flow rate 1.0 mL min^{-1}) was employed, and eluting products were detected by UV at 220 nm. A solvent system consisting of 0.1% TFA aqueous solution (v/v, solvent A) and 0.1% TFA in MeCN (v/v, solvent B) was used for HPLC elution.

Circular Dichroism (CD) Analysis of ubiquitin chain: CD spectra were recorded in a J-820 (JASCO Corporation) instrument. The samples were dissolved in 10 mM phosphate buffer (pH 7.0).

2. Experimental procedure for the synthesis of peptide fragments

Synthesis of Ub (1-45) peptide thioester (1)

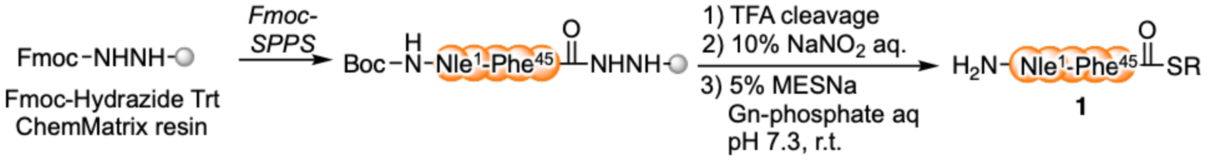

On the Fmoc-hydrazine trityl ChemMatrix® resin (0.31 mmol/g loading), the peptide corresponding ubiquitin sequence [Gln^{2}–Phe^{45}] was elongated by using an automated microwave-assisted synthesis, followed by the the Boc-Nle-OH was coupled at *N*-teminus. The peptidyl resin was washed with DMF×3, DCM×3, MeOH×3, Et_2O×3, and dried under reduced pressure. Dried resin (0.30 g) was cleaved using a solution of TFA/*m*-cresol/thioanisole/TIPS/H_2O (80/5/10/2.5/2.5, (v/v), 50 µL/1.0 mg resin) at room temperature. After 2 h, 10% (w/w) aqueous solution of $NaNO_2$ (1.0 µL/1.0 mg resin) was added to the mixture at -10 °C. Stored at -10 °C for 20 min, cold Et_2O was added to the reaction mixture to give a precipitate. The formed precipitate was collected by centrifugation and thoroughly washed with Et_2O to afford crude peptide azide. To the crude product was added 5% (w/w) MESNa in buffer (6 M Gn·HCl, 0.2 M Na phosphate, pH 7.3). After 2 h at room temperature, TFA was added to quench the thiolysis (pH < 3), analyzed and purified by reversed-phase HPLC (30–36% solvent B over 60 min, 0.1% TFA, YMC-Triart C8 20×250 mm column) followed by lyophilization to yield peptide **1** (6.9 µmol, 14% yield). Analytical HPLC, t_R = 9.2 min (30–36%B over 30 min, 0.1% TFA, COSMOSIL 5C$_8$-AR300 4.6×250 mm column); HRMS (ESI), Calcd for $C_{232}H_{387}N_{58}O_{73}S_2$ $[M+H]^+$ 5221.1005, found 5220.8168.

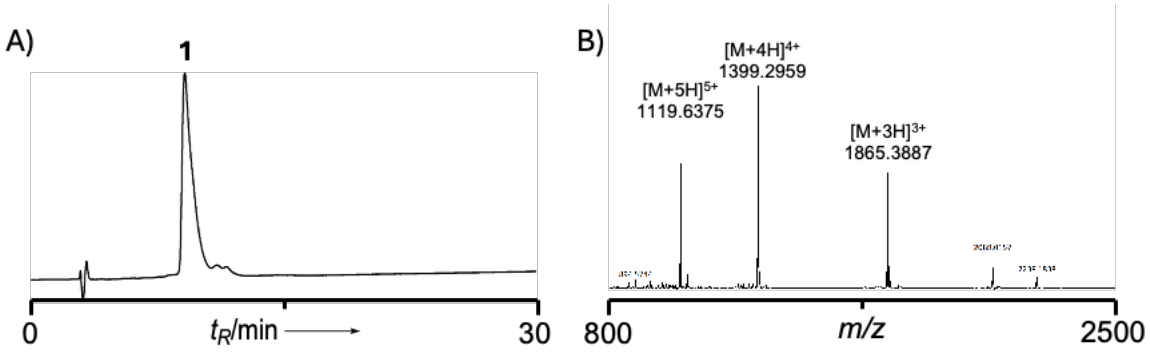

Figure S1: A) HPLC-trace and B) ESI-MS spectrum of purified peptide thioester **1.**

Synthesis of Dap20-linked branched peptide (2)

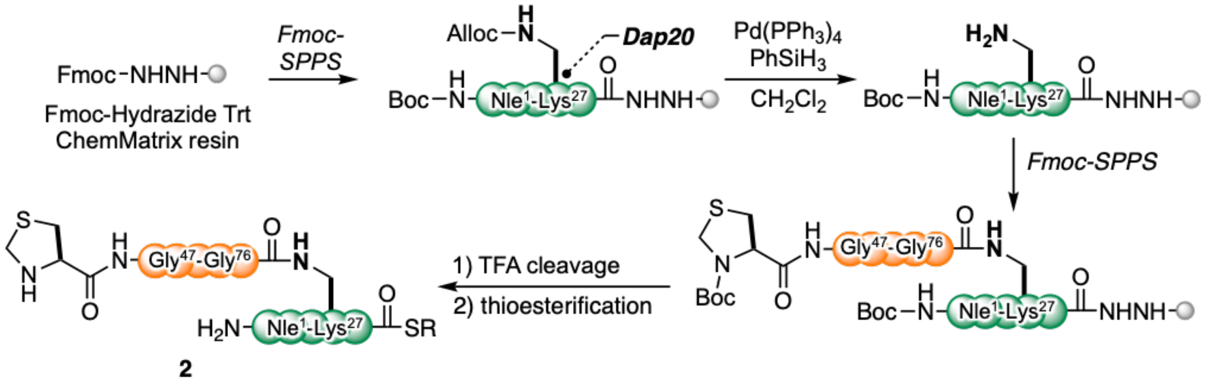

On the Fmoc-hydrazine trityl ChemMatrix® resin (0.31 mmol/g loading), the peptide corresponding ubiquitin sequence [Asp^{21}–Lys^{27}] was elongated by using an automated microwave-assisted synthesis and manual synthesis (4.0 eq. each of amino acid using HATU (3.8 eq.) and DIPEA (4.0 eq.) in DMF and Fmoc removal with 20% piperidine in DMF (10 min)). Sequentially, Fmoc-Dap(Alloc)-OH (4.0 eq.) was coupled manually using DIPCI (4.0 eq.) and Oxyma in DMF for 1 h, followed by manual Fmoc-SPPS to couple the next peptide sequence [Gln^{2}–Glu^{18}] was elongated and the Boc-Nle-OH was coupled at *N*-teminus. Then, $Pd(PPh_3)_4$ (0.25 eq.) and $PhSiH_3$ (20.0 eq.) in CH_2Cl_2 was applied to remove the Alloc group, followed by the peptide sequence [Gly^{47}–Gly^{75}] was elongated and Boc-Thz-OH was coupled at *N*-terminus. The peptidyl resin was washed with DMF×3, DCM×3, MeOH×3, Et_2O×3, and dried under reduced pressure. Dried resin (0.80 g) was cleaved using a solution of TFA/*m*-cresol/thioanisole/TIPS/H_2O (80/5/10/2.5/2.5, (v/v), 50 µL/1.0 mg resin) at room temperature. After 2 h, 10% aqueous solution of $NaNO_2$ (1.0 µL/1.0 mg resin) was added to the mixture at -10 °C. Stored at -10 °C for 20 min, cold Et_2O was added to the reaction mixture to give a precipitate. The formed precipitate was collected by centrifugation and thoroughly washed with Et_2O to afford crude peptide azide. To the crude product was added 5% (w/w) MESNa in buffer (6 M Gn·HCl, 0.2 M Na phosphate, pH 7.3). After 2 h at room temperature, TFA was added to quench the thiolysis (pH < 3), analyzed and purified by reversed-phase HPLC (28–38% solvent B over 60 min, 0.1% TFA, COSMOSIL 5C_{18}-AR-II 20×250 mm column) followed by lyophilization to yield peptide **2** (2.7 µmol, 3% yield). Analytical HPLC, t_R = 13.2 min (28–38%B in 30 min, 0.1% TFA, COSMOSIL 5C_{18}-AR-Ⅱ 4.6×250 mm column); HRMS (ESI), Calcd for $C_{288}H_{484}N_{80}O_{92}S_3$ $[M+H]^+$ 6636.6955, found 6636.1643.

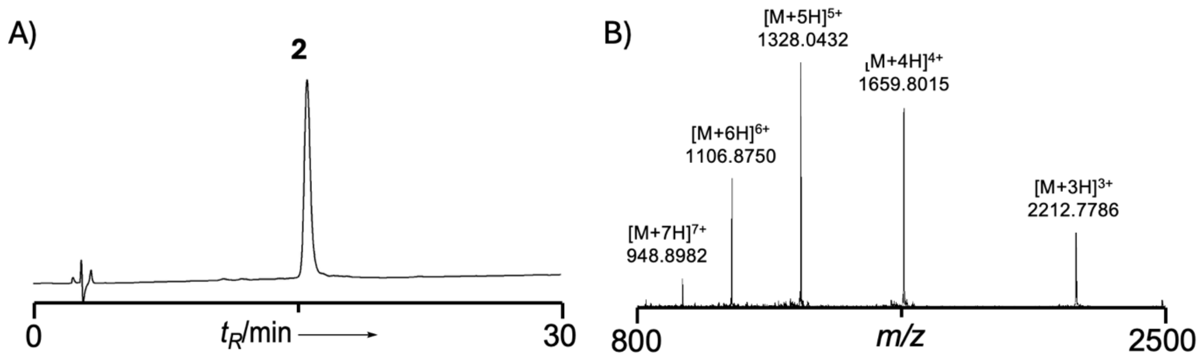

Figure S2: A) HPLC-trace and B) ESI-MS spectrum of purified peptide thioester **2.**

Synthesis of Ser20-linked branched peptide (3)

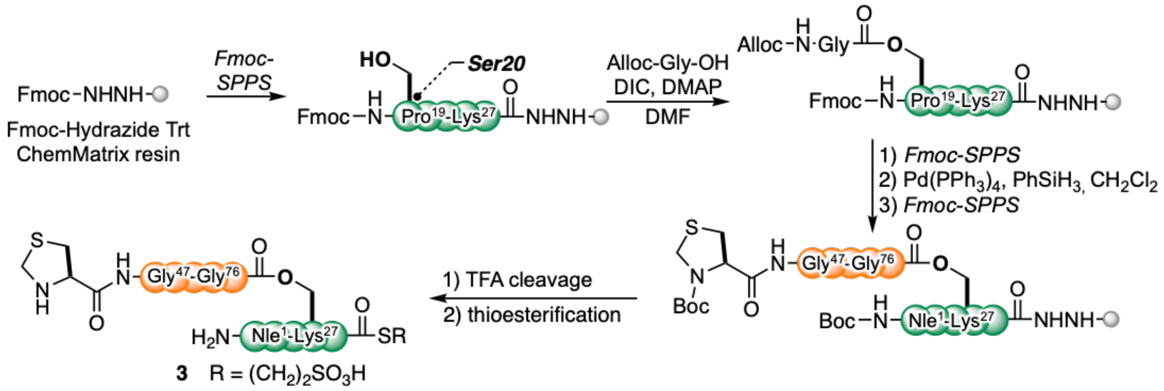

On the Fmoc-hydrazine trityl ChemMatrix® resin (0.31 mmol/g loading), the peptide corresponding ubiquitin sequence [Asp^{21}–Lys^{27}] was elongated by using an automated microwave-assisted synthesis and manual synthesis (4.0 eq. each of amino acid using HATU (3.8 eq.) and DIPEA (4.0 eq.) in DMF and Fmoc removal with 20% piperidine in DMF (10 min)). Sequentially, Fmoc-Ser(OH)-OH (4.0 eq.) and Fmoc-Pro-OH (4.0 eq.) were coupled manually using DIPCI (4.0 eq.) and Oxyma (3.0 eq.) in DMF for 1 h, followed by Alloc-Gly-OH (10 eq.) was introduced to the unprotected Ser20 with DIPCI (10 eq.) and DMAP (2.0 eq.) in DMF for 2h. The next peptide sequence [Gln^{2}–Glu^{18}] was elongated and the Boc-Nle-OH was coupled at *N*-teminus. Then, $Pd(PPh_3)_4$ (0.25 eq.) and $PhSiH_3$ (20.0 eq.) in CH_2Cl_2 was applied to remove the Alloc group, followed by the peptide sequence [Gly^{47}–Gly^{75}] was elongated and Boc-Thz-OH was coupled at *N*-terminus. The peptidyl resin was washed with DMF×3, DCM×3, MeOH×3, Et_2O×3, and dried under reduced pressure. Dried resin (0.50 g) was cleaved using a solution of TFA/*m*-cresol/thioanisole/TIPS/H_2O (80/5/10/2.5/2.5, (v/v), 50 µL/1.0 mg resin) at room temperature. After 2 h, 10% (w/w) aqueous solution of $NaNO_2$ (1.0 µL/1.0 mg resin) was added to the mixture at -10 °C. Stored at -10 °C for 20 min, cold Et_2O was added to the reaction mixture to give a precipitate. The formed precipitate was collected by centrifugation and thoroughly washed with Et_2O to afford crude peptide azide. To the crude product was added 5% (w/w) MESNa in buffer (6 M Gn·HCl, 0.2 M Na phosphate, pH 7.3). After 6 h at room temperature, TFA was added to quench the thiolysis (pH < 3), analyzed and purified by reversed-phase HPLC (25–35% solvent B over 60 min, 0.1% TFA, COSMOSIL $5C_{18}$-AR-II 20×250 mm column) followed by lyophilization to yield peptide **3** (1.0 µmol, 1% yield). Analytical HPLC, t_R = 24.2 min (25–35%B in 30 min, 0.1% TFA, COSMOSIL $5C_{18}$-AR-II 4.6×250 mm column); HRMS (ESI), Calcd for $C_{288}H_{484}N_{79}O_{93}S_3$ $[M+H]^+$ 6637.6795, found 6637.2362.

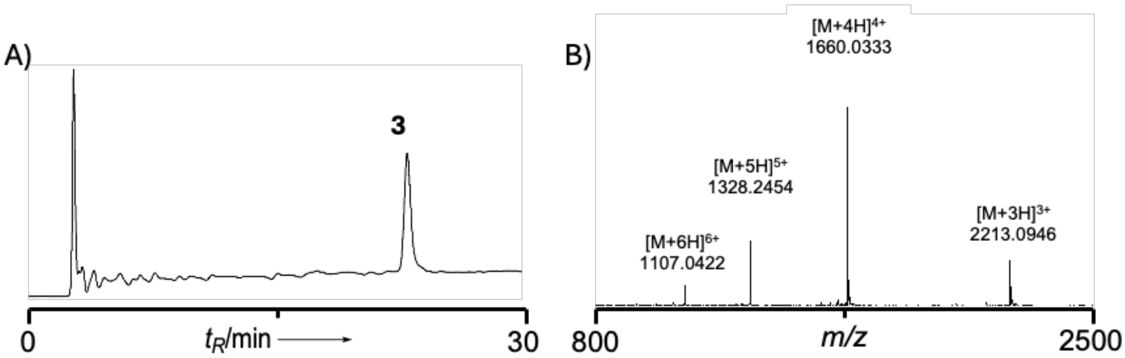

Figure S3: A) HPLC-trace and B) ESI-MS spectrum of purified peptide thioester **3.**

Synthesis of *N*-terminal cysteinyl Ub (28-76) peptide (4)

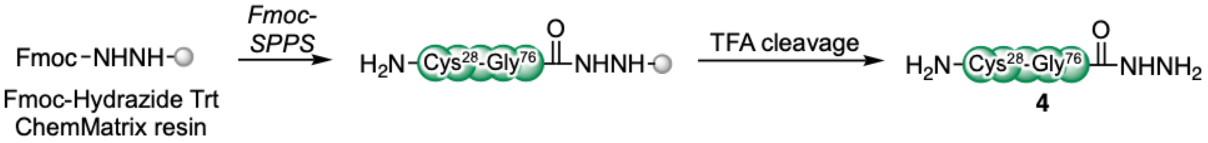

On the Fmoc-hydrazine trityl ChemMatrix® resin (0.31 mmol/g loading), the peptide corresponding ubiquitin sequence [Cys^{28}–Gly^{76}] was elongated by using a manual synthesis. The peptidyl resin was washed with DMF×3, DCM×3, MeOH×3, Et_2O×3, and dried under reduced pressure. Dried resin (0.78 g) was cleaved using a solution of TFA/TIPS/H_2O (95/2.5/2.5, (v/v), 50 µL/1.0 mg resin) at room temperature. After 2 h, The resin in the reaction mixture was removed by filtration and then the filtrate was concentrated in a stream of nitrogen gas. To the resulting filtrate was added cold Et_2O and the formed precipitate was collected by centrifugation to afford crude peptide. The crude peptide was purified by reversed-phase HPLC (28–38% solvent B over 60 min, 0.1% TFA, COSMOSIL 5C_{18}-AR-II 20×250 mm column) followed by lyophilization to yield peptide **4** (6.2 µmol, 6% yield). Analytical HPLC, t_R = 15.4 min (23–33%B in 30 min, 0.1% TFA, COSMOSIL 5C_{18}-AR-II 4.6×250 mm column); HRMS (ESI), Calcd for $C_{243}H_{406}N_{75}O_{74}S$ $[M+H]^+$ 5594.4315, found 5594.1250.

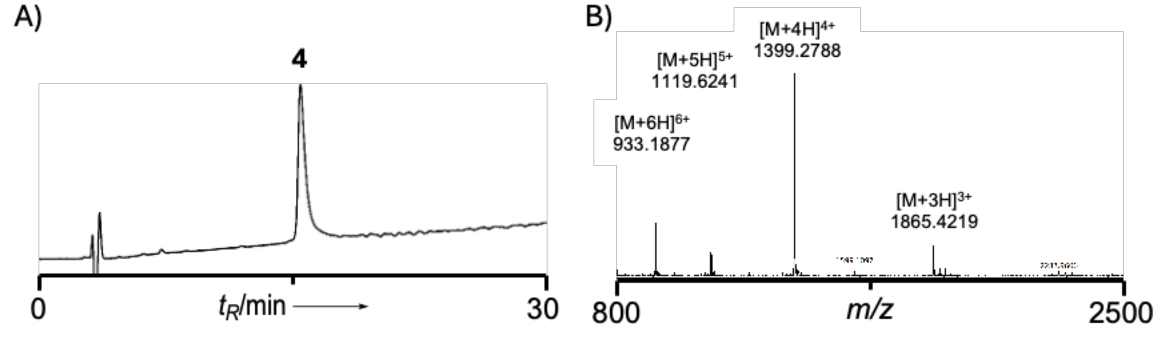

Figure S4: A) HPLC-trace and B) ESI-MS spectrum of purified peptide **4.**

Synthesis of *N*-terminal cysteinyl biotinylated peptide (5)

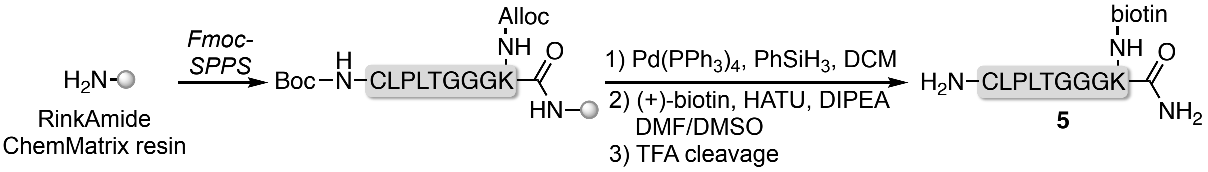

On the RinkAmide ChemMatrix® resin (0.47 mmol/g loading), the peptide sequence [Boc-CLPLTGGGK] with Alloc protection at ε-NH_2 was elongated by using an automated microwave-assisted synthesis. Alloc group was deprotected using $Pd(PPh_3)_4$ (0.25 eq.) and $PhSiH_3$ (20 eq.), followed by (+)-biotin (6.0 eq.) was coupled with HATU (5.7 eq.), DIPEA (6.0 eq.) in DMF/DMSO (50/50 (v/v)) for 2 h. The peptidyl resin was washed with DMF×3, DCM×3, MeOH×3, Et_2O×3, and dried under reduced pressure. Dried resin (0.13 g) was cleaved using a solution of TFA/TIPS/H_2O (95/2.5/2.5, (v/v), 50 µL/1.0 mg resin) at room temperature. After 2 h, The resin in the reaction mixture was removed by filtration and then the filtrate was concentrated in a stream of nitrogen gas. To the resulting filtrate was added cold Et_2O and the formed precipitate was collected by centrifugation to afford crude peptide. The crude peptide was purified by reversed-phase HPLC (18–27% solvent B over 60 min, 0.1% TFA, COSMOSIL

5C18-AR-II 20×250 mm column) followed by lyophilization to yield peptide **5** (8.4 µmol, 17% yield). Analytical HPLC, t_R = 13.9 min (18–27%B in 30 min, 0.1% TFA, COSMOSIL 5C18-AR-II 4.6×250 mm column); HRMS (ESI), Calcd for $C_{46}H_{80}N_{13}O_{12}S_2$ $[M+H]^+$ 1070.5485, found 1070.5582.

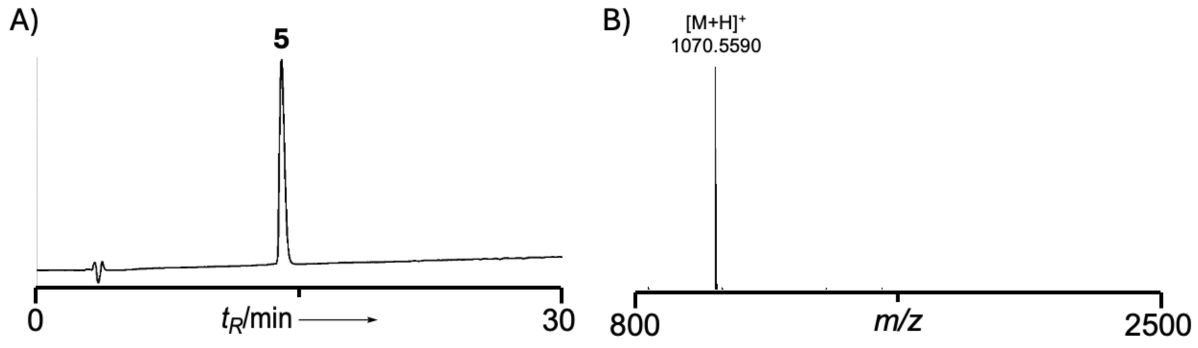

Figure S5: a) HPLC-trace and b) ESI-MS spectrum of purified peptide **5.**

Synthesis of Ub (1-45) peptide thioester (6)

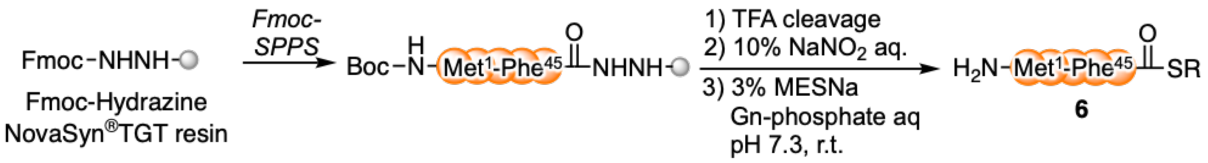

On Fmoc-hydrazine trityl ChemMatrix® resin (0.22 mmol/g loading), the peptide corresponding ubiquitin sequence [Gln²–Phe⁴⁵] was elongated by using an automated microwave-assisted synthesis, followed by the the Boc-Met-OH was coupled at *N*-teminus. The peptidyl resin was washed with DMF×3, DCM×3, MeOH×3, Et_2O×3, and dried under reduced pressure. Dried resin (0.52 g) was cleaved using a solution of TFA/*m*-cresol/thioanisole/TIPS/H_2O (80/5/10/2.5/2.5, (v/v), 50 µL/1.0 mg resin) at 37 ºC. After 2 h, 10% (w/w) aqueous solution of $NaNO_2$ (1.0 µL/1.0 mg resin) was added to the mixture at -10 °C. Stored at -10 °C for 30 min, cold Et_2O was added to the reaction mixture to give a precipitate. The formed precipitate was collected by centrifugation and thoroughly washed with Et_2O to afford crude peptide azide. To the crude product was added 3% (w/w) MESNa in buffer (6 M Gn·HCl, 0.2 M Na phosphate, pH 7.3). After 1 h at room temperature, TFA was added to quench the thiolysis (pH < 3), analyzed and purified by reversed-phase HPLC (28–48% solvent B over 60 min, 0.1% TFA, COSMOSIL 5C8 AR-300 20×250 mm column) followed by lyophilization to yield peptide **6** (9.1 µmol, 14% yield). Analytical HPLC, t_R = 5.7 min (31% solvent B over 15 min, 0.1% TFA, COSMOSIL 5C8-AR300 4.6×250 mm column); HRMS (ESI), Calcd for $C_{231}H_{385}N_{58}O_{73}S_3$ $[M+H]^+$ 5239.1335, found 5238.8866.

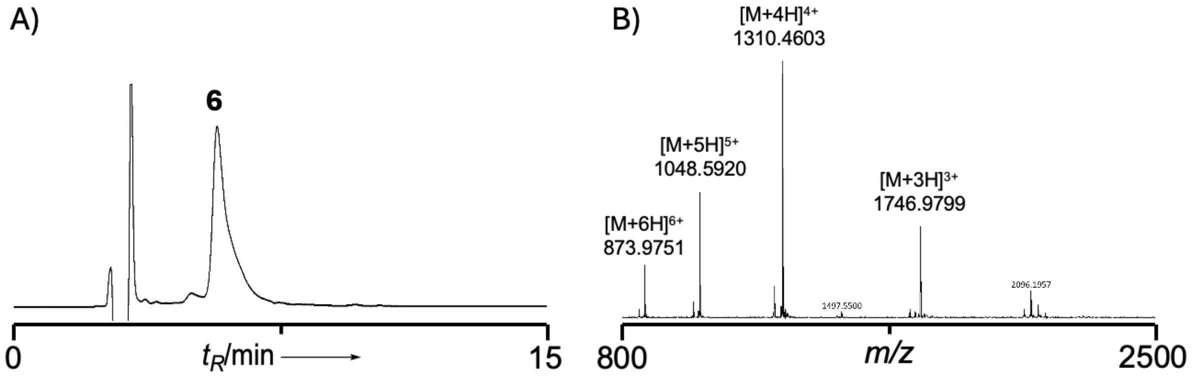

Figure S6: A) HPLC-trace and B) ESI-MS spectrum of purified peptide thioester **6.**

Synthesis of Ser20-linked branched peptide (7)

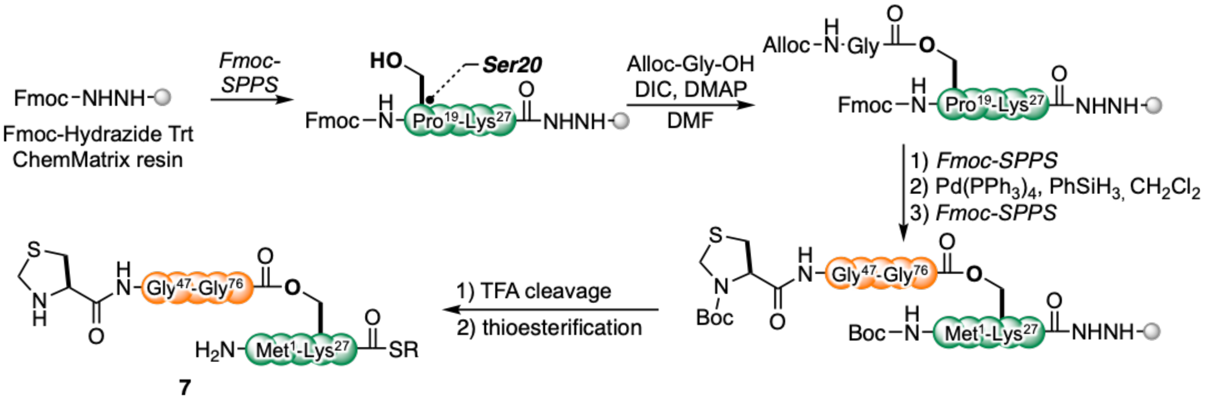

On the Fmoc-hydrazine NovaSyn®TGT resin (0.20 mmol/g loading), the peptide corresponding ubiquitin sequence [Asp^{21}–Lys^{27}] was elongated by using an automated microwave-assisted synthesis and manual synthesis (3.0 eq. each of amino acid using HATU (2.9 eq.) and DIPEA (4.0 eq.) in DMF and Fmoc removal with 20% piperidine in DMF (5.0 min)). Sequentially, Fmoc-Ser(OH)-OH (3.0 eq.) and Fmoc-Pro-OH (3.0 eq.) were coupled manually using DIPCI (3.0 eq.) and Oxyma (3.0 eq.) in DMF for 1 h, followed by Alloc-Gly-OH (10 eq.) was introduced to the unprotected Ser20 with DIPCI (10 eq.) and DMAP (2.0 eq.) in DMF for 2h. The next peptide sequence [Gln^{2}–Glu^{18}] was elongated and the Boc-Met-OH was coupled at *N*-teminus. Then, $Pd(PPh_3)_4$ (0.25 eq.) and $PhSiH_3$ (20.0 eq.) in CH_2Cl_2 was applied to remove the Alloc group, followed by the peptide sequence [Gly^{47}–Gly^{75}] was elongated and Boc-Thz-OH was coupled at *N*-terminus. The peptidyl resin was washed with DMF×3, DCM×3, MeOH×3, Et_2O×3, and dried under reduced pressure. Dried resin (0.72 g) was cleaved using a solution of TFA/*m*-cresol/thioanisole/TIPS/H_2O (80/5/10/2.5/2.5, (v/v), 50 µL/1.0 mg resin) at room temperature. After 2 h, 10% (w/w) aqueous solution of $NaNO_2$ (1.0 µL/1.0 mg resin) was added to the mixture at -10 °C. Stored at -10 °C for 20 min, cold Et_2O was added to the reaction mixture to give a precipitate. The formed precipitate was collected by centrifugation and thoroughly washed with Et_2O to afford crude peptide azide. To the crude product was added 3% (w/w) MESNa in buffer (6 M Gn·HCl, 0.2 M Na phosphate, pH 7.2). After 6 h at room temperature, TFA was added to quench the thiolysis (pH < 3), analyzed and purified by reversed-phase HPLC (28–38% solvent B over 60 min, 0.1% TFA, COSMOSIL Protein-R 20×250 mm column) followed by lyophilization to yield peptide **7** (1.3 µmol, 2% yield). Analytical UHPLC, t_R = 3.06 min (28-38%B in 5.0 min, 0.1% TFA, JASCO Unifinepak 3.0×50

mm column); HRMS (ESI), Calcd for $C_{287}H_{482}N_{79}O_{93}S_4$ $[M+H]^+$ 6655.7125, found 6655.4724.

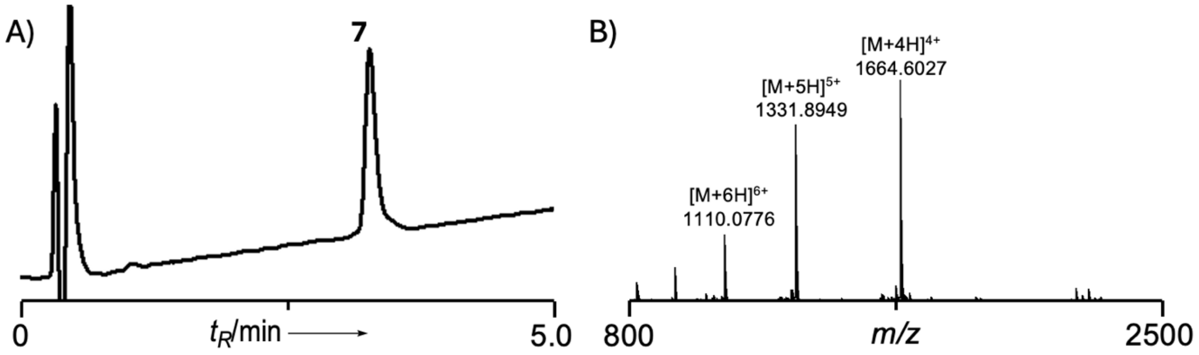

Figure S7: A) HPLC-trace and B) ESI-MS spectrum of purified peptide thioester **7.**

Synthesis of *N*-terminal cysteinyl Ub (28-76) peptide with biotin (8)

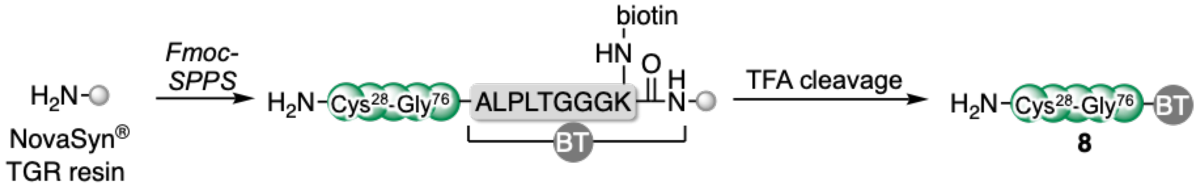

On the NovaSyn®TGR resin (0.22 mmol/g loading), the peptide sequence [ALPLTGGGK], biotinylated at the ε-NH_2 group, was elongated, followed by elongation with the ubiquitin-derived sequence [Cys28–Gly76]. The peptidyl resin was washed with DMF×3, DCM×3, MeOH×3, Et_2O×3, and dried under reduced pressure. Dried resin (0.85 g) was cleaved using a solution of TFA/TIPS/H_2O (95/2.5/2.5, (v/v), 50 µL/1.0 mg resin) at room temperature. After 2 h, The resin in the reaction mixture was removed by filtration and then the filtrate was concentrated in a stream of nitrogen gas. To the resulting filtrate was added cold Et_2O and the formed precipitate was collected by centrifugation to afford crude peptide. The crude peptide was purified by reversed-phase HPLC (28–38% solvent B over 60 min, 0.1% TFA, COSMOSIL Protein-R 20×250 mm column) followed by lyophilization to yield peptide **8** (3.4 µmol, 3% yield). Analytical UHPLC, t_R = 2.40 min (28–38%B in 5.0 min, 0.1% TFA, JASCO Unifinepak 3.0×50 mm column); HRMS (ESI), Calcd for $C_{289}H_{481}N_{86}O_{86}S_2$ $[M+H]^+$ 6600.6625, found 6600.2983.

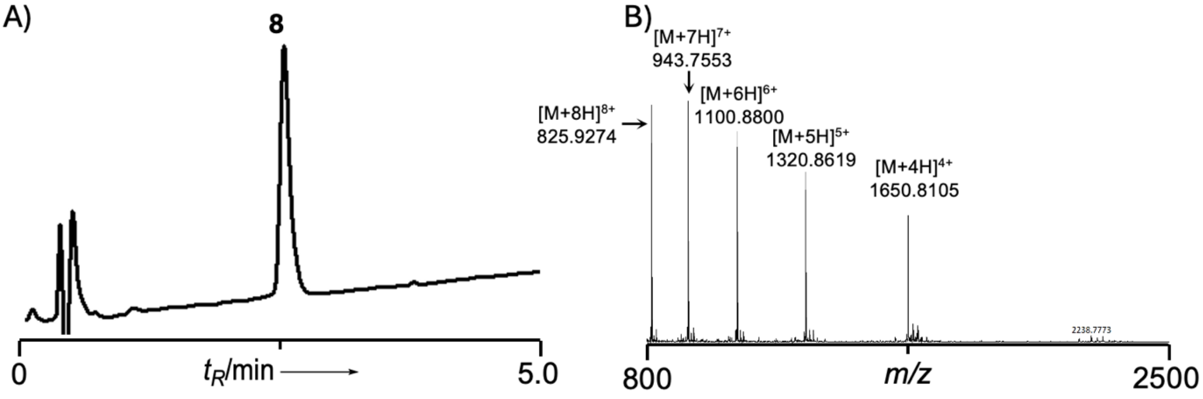

Figure S8: A) HPLC-trace and B) ESI-MS spectrum of purified peptide **8.**

3. Experimental procedure for peptide ligation and desulfurization

Synthesis of Dap20-linked diUb precursor (11)

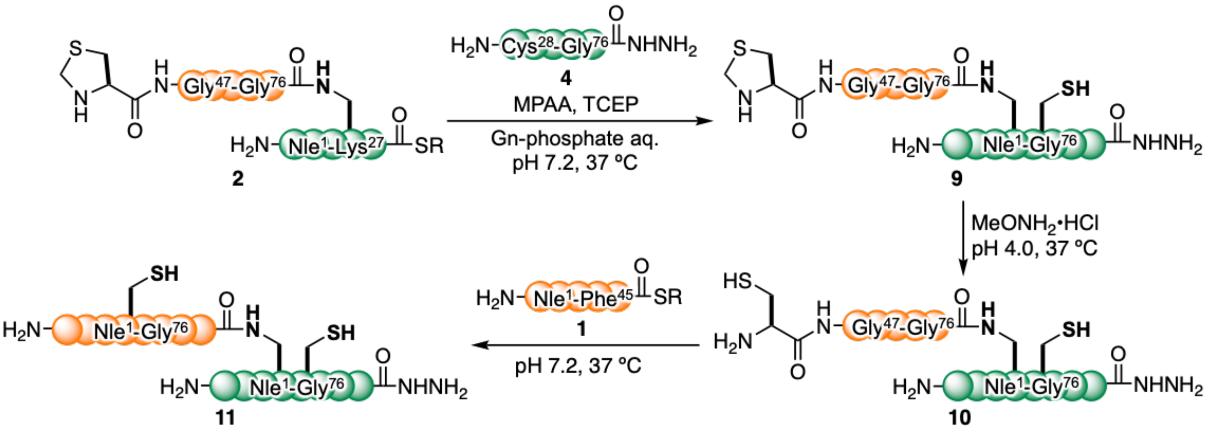

The lyophilized peptide **2** (1.0 μmol) and peptide **4** (1.1 μmol) were dissolved under nitrogen atmosphere in degassed buffer (100 mM MPAA, 50 mM TCEP, 6 M Gn•HCl, 200 mM phosphate, pH 7.2) to a final concentration of 1.0 mM. The reaction mixture was incubated at 37 ºC for 2 h. To monitor the progress of the reaction, aliquots were withdrawn from the ligation mixture, quenched with an aqueous solution of 6 M Gn•HCl in 0.1% TFA aq. and analyzed by HPLC. After completion of the peptide ligation, to the reaction mixture was added $MeONH_2 \cdot HCl$ to a concentration of 200 mM to remove the Thz group. Then, the pH was adjusted to neutral and to the reaction mixture was added peptide **1** (1.3 μmol) in buffer to a final concentration of 0.50 mM at 37 ºC for 3 h. The crude peptide was purified by preparative reverse phase HPLC (25–40% solvent B over 60 min, 0.1% TFA, YMC-Triart C8 10×250 mm column) followed by lyophilization to yield peptide **11** (88 nmol, 9% yield). Analytical HPLC, t_R = 24.8 min (25–40%B in 30 min, 0.1% TFA, COSMOSIL Protein-R 4.6×250 mm column); HRMS (ESI), Calcd for $C_{758}H_{1264}N_{213}O_{233}S_2$ $[M+H]^+$ 17153.8275, found 17153.4056.

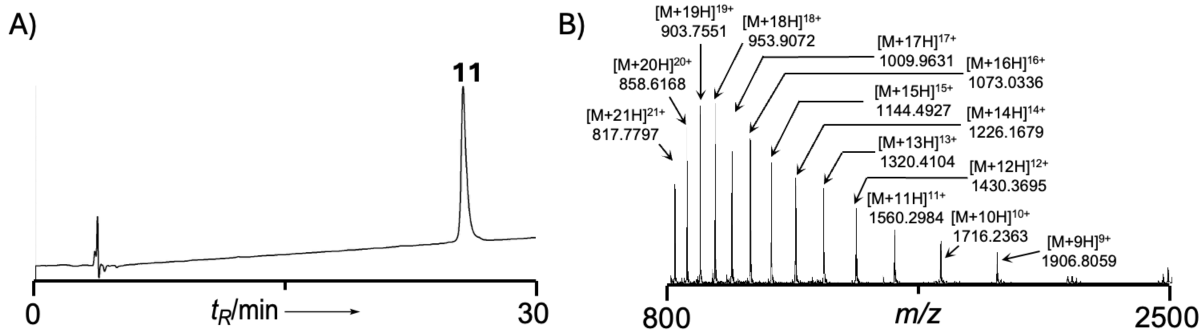

Figure S9: a) HPLC-trace and b) ESI-MS spectrum of purified protein **11**.

Synthesis of biotinylated Dap20-linked diUb (14)

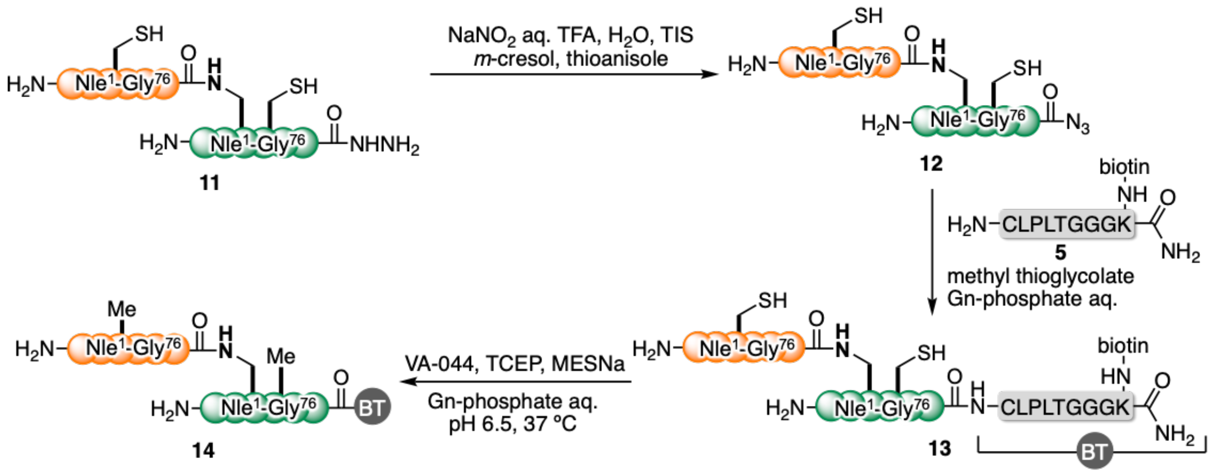

The lyophilized Dap20-linked diUb precursor **11** (40 nmol) was dissolved in TFA-*m*-cresol-thioanisole-isopropylsilane-H_2O (80:5:10:2.5:2.5 (v/v), 77 μL) at -10°C and then 10% (*w*/*w*) $NaNO_2$ aq (7.3 eq) was added to the mixture. Stored at -10 °C for 20 min, cold Et_2O was added to the reaction mixture to give a precipitate. The formed precipitate was collected by centrifugation and thoroughly washed with Et_2O. Then the precipitate and biotinylated peptide **5** (80 nmol) was dissolved in buffer (100 mM methyl thioglycolate, 6 M Gn·HCl, 200 mM phosphate, pH 7.0) (300 μL) to a final concentration 0.13 mM. The reaction was incubated at 37 ºC for 6 h. Then the mixture (300 uL) was subjected for the desulfurization by treating it with TCEP (500 mM), MESNa (200 mM) and VA- 044 (50 mM) to a final concentration 65 nM at 37 ºC for 24 h. The progress of the reaction was monitored using analytical reverse phase HPLC with the gradient of 25-40% B over 30 min. The product was purified by preparative reverse phase HPLC (25–40% solvent B over 60 min, 0.1% TFA, Protein-R 10×250 mm column) followed by lyophilization to yield the biotinylated Dap20-linked diUb **14** (5.2 nmol, 13 % yield). Analytical HPLC, t_R = 25.2 min (25–40%B in 30 min, 0.1% TFA, COSMOSIL Protein-R 4.6×250 mm column); HRMS (ESI), Calcd for $C_{804}H_{1339}N_{224}O_{245}S$ $[M+H]^+$ 18095.9385, found 18095.7506.

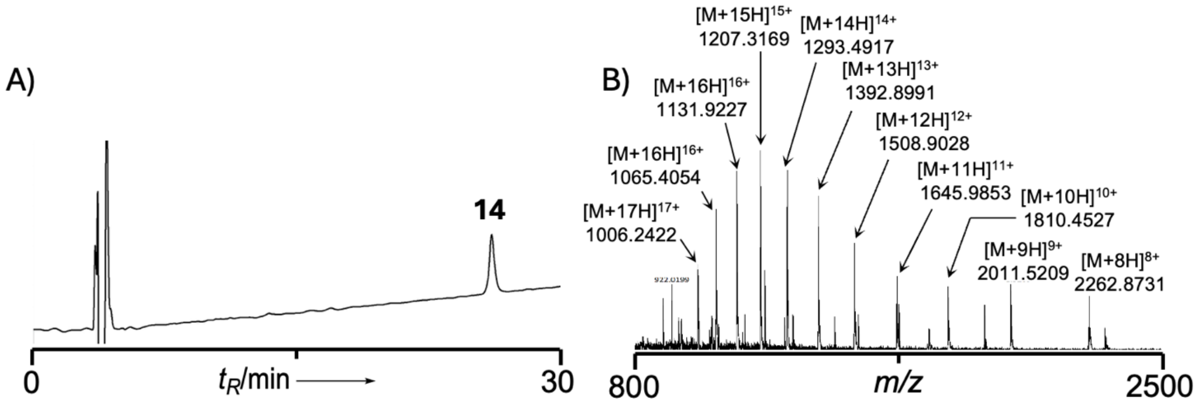

Figure S10: a) HPLC-trace and b) ESI-MS spectrum of purified protein **14**.

Synthesis of Ser20-linked diUb precursor (17)

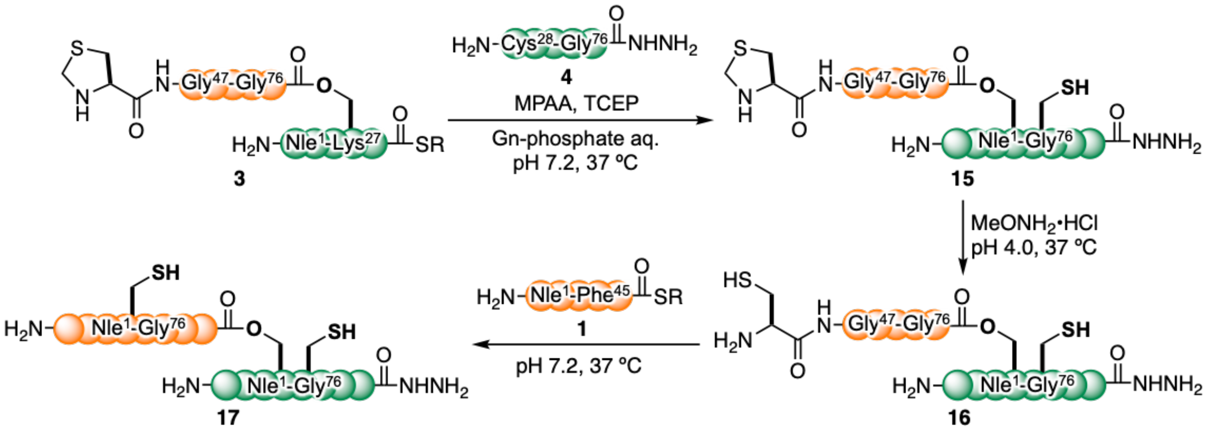

The lyophilized peptide **3** (0.82 µmol) and peptide **4** (0.90 µmol) were dissolved under nitrogen atmosphere in degassed buffer (40 mM MPAA, 20 mM TCEP, 6.0 M Gn•HCl, 0.20 M phosphate, pH 7.2) to a final concentration of 1.0 mM. The reaction mixture was incubated at 37 ºC for 2 h. To monitor the progress of the reaction, aliquots were withdrawn from the ligation mixture, quenched with an aqueous solution of 6.0 M Gn·HCl in 0.1% TFA aq. and analyzed by HPLC. After completion of the peptide ligation, to the reaction mixture was added $MeONH_2$ ·HCl to a concentration of 0.20 M to remove the Thz group. Then, the pH was adjusted to neutral and to the reaction mixture was added peptide **1** (1.1 µmol) in buffer to a final concentration of 0.50 mM at 37 ºC for 4 h. The crude peptide was purified by preparative reverse phase HPLC (25–40% solvent B over 60 min, 0.1% TFA, YMC-Triart C8 10×250 mm column) followed by lyophilization to yield peptide **17** (88 nmol, 11% yield). Analytical HPLC, t_R = 24.8 min (25–40%B in 30 min, 0.1% TFA, COSMOSIL Protein-R 4.6×250 mm column); HRMS (ESI), Calcd for $C_{758}H_{1264}N_{212}O_{234}S_2$, $[M+H]^+$ 17154.8115, found 17154.8620.

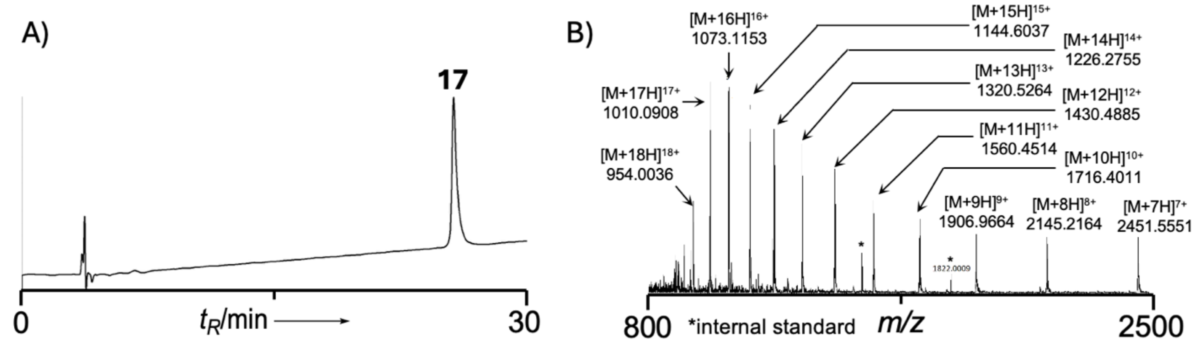

Figure S11: a) HPLC-trace and b) ESI-MS spectrum of purified peptide **17**.

Synthesis of biotinylated Ser20-linked diUb precursor (20)

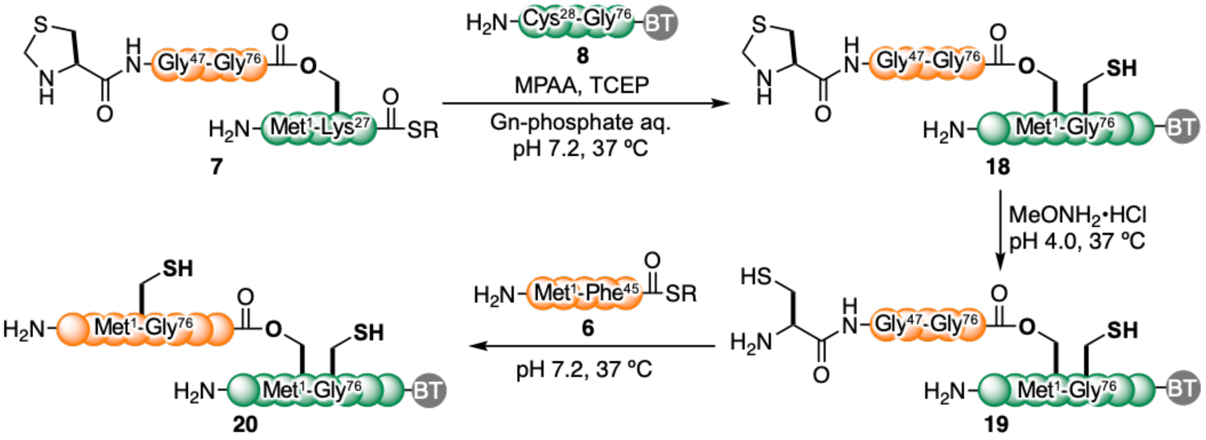

The lyophilized peptide **7** (1.0 µmol) and peptide **8** (1.1 µmol) were dissolved under nitrogen atmosphere in degassed buffer (100 mM MPAA, 50 mM TCEP, 6.0 M Gn·HCl, 0.20 M phosphate, pH 7.2) to a final concentration of 1.0 mM. The reaction mixture was incubated at 37 ºC for 2 h. To monitor the progress of the reaction, aliquots were withdrawn from the ligation mixture, quenched with an aqueous solution of 6.0 M Gn·HCl in 0.1% TFA aq. and analyzed by HPLC. After completion of the peptide ligation, to the reaction mixture was added $MeONH_2$ ·HCl to a concentration of 0.20 M to remove the Thz group. Then, the pH was adjusted to neutral and to the reaction mixture was added peptide **6** (1.1 µmol) in buffer to a final concentration of 0.50 mM at 37 ºC for 4 h. The crude peptide was purified by preparative reverse phase HPLC (28–43% solvent B over 60 min, 0.1% TFA, Protein-R 10×250 mm column) followed by lyophilization to yield peptide **20** (99 nmol, 10% yield). Analytical HPLC, t_R = 20.2 min (28–43%B in 30 min, 0.1% TFA, COSMOSIL Protein-R 4.6×250 mm column); HRMS (ESI), Calcd for $C_{802}H_{1334}N_{223}O_{246}S_5$, $[M+H]^+$ 18197.1085, found 18197.0374.

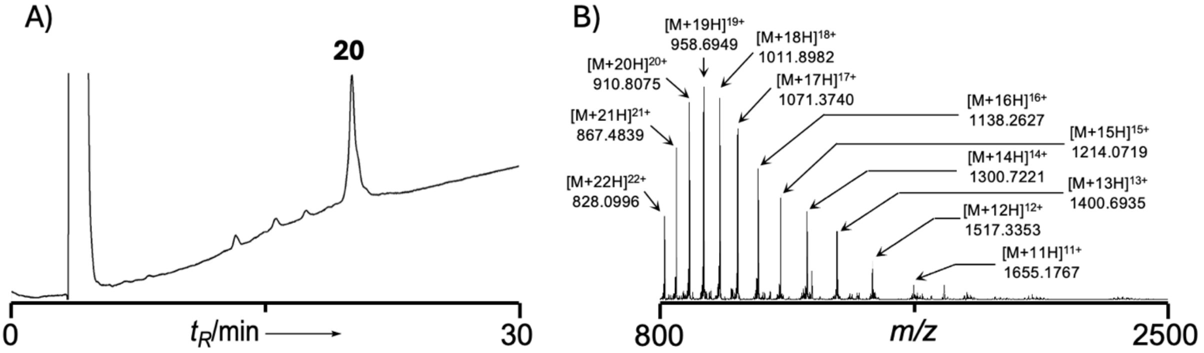

Figure S12: a) HPLC-trace and b) ESI-MS spectrum of purified protein **20**.

Synthesis of biotinylated Ser20-linked diUb (21)

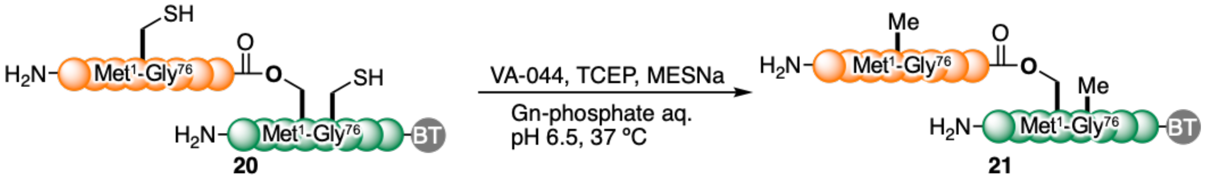

The lyophilized diUb precursor **20** (99 nmol) was dissolved under nitrogen atmosphere in degassed buffer (50 mM VA-044, 500 mM TCEP, 200 mM MESNa, 6 M Gn·HCl, 200 mM

pshosphate at pH 6.5) to a final concentration 0.25 mM. The reaction was incubated at 37 ºC for 32 h. The progress of the reaction was monitored using analytical HPLC. The product **21** was purified by preparative reverse phase HPLC (28–43% solvent B over 60 min, 0.1% TFA, Protein-R 10×250 mm column) followed by lyophilization to yield the biotinylated Ser20-linked diUb **21** (24 nmol, 24 % yield). Analytical HPLC, t_R = 15.2 min (28–43%B in 30 min, 0.1% TFA, COSMOSIL 5C18-AR-II 4.6×250 mm column); HRMS (ESI), Calcd for $C_{802}H_{1334}N_{223}O_{246}S_3$ $[M+H]^+$ 18132.9885, found 18132.7218.

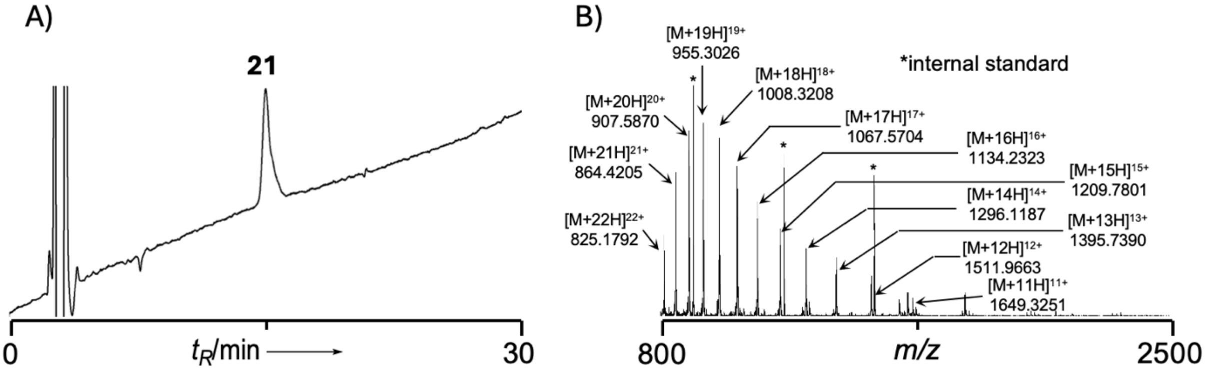

Figure S13: a) HPLC-trace and b) ESI-MS spectrum of purified protein **21**.

Chemical stability of diUb in desulfurization

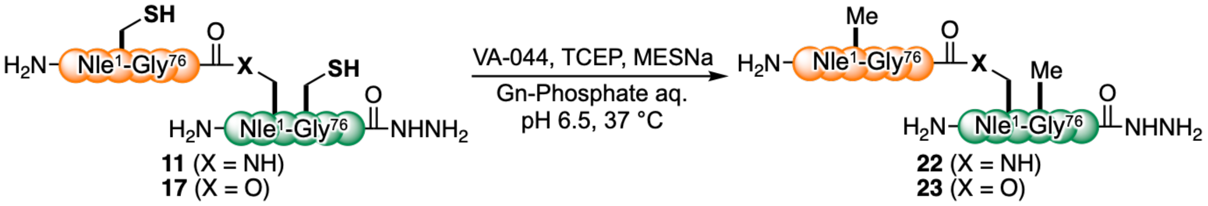

The lyophilized diUb precursor **11** or **17** was dissolved under nitrogen atmosphere in degassed buffer (50 mM VA-044, 500 mM TCEP, 200 mM MESNa, 6 M Gn·HCl, 200 mM pshosphate at pH 6.5) to a final concentration 0.10 mg/ml. The reaction was incubated at 37 ºC for 22 h. The progress of the reaction was monitored using analytical HPLC (25–40% B over 30 min, 0.1% TFA, COSMOSIL Protein-R 4.6×250 mm column).

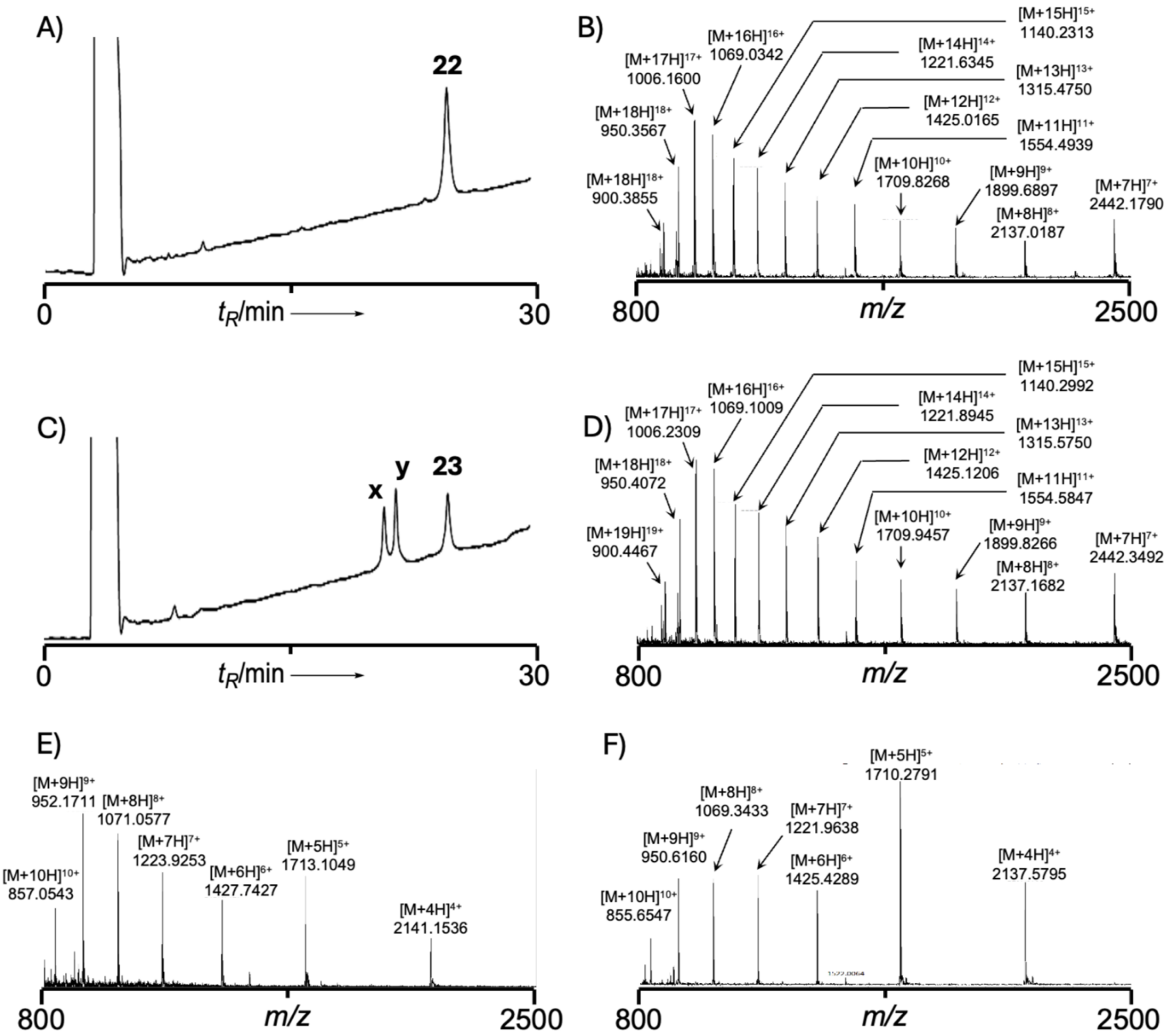

Figure S14: A) HPLC-trace of desulfurization of Dap-linked diUb precursor **11**. B) ESI-MS spectrum of the peak **22** with observed mass $[M+H]^+$ 17089.3804 (calcd for $C_{758}H_{1263}N_{213}O_{233}$ $[M+H]^+$ 17089.7075). C) HPLC-trace of desulfurization of Ser-linked diUb precursor **17**. D) ESI-MS spectrum of the peak **23** with observed mass $[M+H]^+$ 17090.2376 (calcd for $C_{758}H_{1263}N_{212}O_{234}$ $[M+H]^+$ 17090.6915). E) ESI-MS spectrum of the peak **x** corresponds to Ub-NHNH$_2$ with observed mass $[M+H]^+$ 8561.4754 (calcd for $C_{379}H_{634}N_{107}O_{117}$ $[M+H]^+$ 8561.8725). F) ESI-MS spectrum of the peak **y** corresponds to Ub-OH with observed mass $[M+H]^+$ 8547.4606 Da (calcd for $C_{379}H_{632}N_{105}O_{118}$ $[M+H]^+$ 8547.8415).

4. Experimental procedure of interactome analysis

Plasmid constructs and protein purification: To generate biotinylated ubiquitin enzymatically, we first constructed pET30b Sortase and pET26b Ub-sorHis, which encode Sortase (clone 5Y, aa 60-206, codon-optimized for bacterial expression [Eurofins Genomics])[1-3] and human ubiquitin fused with a Sortase recognition sequence and a 6×His-tag (ALPLTGGHHHHHH) at the C-terminus. Recombinant Sortase and Ub-sorHis were expressed in *E. coli* BL21 (DE3) and purified on TALON resin (Clontech) for further ubiquitination and biotinylation as described below. GST-NPLOC4 and GST-TOLLIP were expressed in *E. coli* BL21 (DE3) harboring pGEX6p-1 NPLOC4 or pGEX6p-1 TOLLIP and purified using Glutathione Sepharose 4B (GE Healthcare). cDNA encoding human USP39 was cloned into pEU-E01-GST, and GST-USP39 was expressed using wheat cell-free protein synthesis system (CellFree Sciences Co) according to the manufacturer's instruction, followed by purification on Glutathione Sepharose 4B. Purified Usp2cc and enzymes for ubiquitination (E1, E2-25K, Ubc13, and MMS2) were described previously.[4,5]

Generation of Lys48- and Lys63-ubiquitin chains: For the synthesis of Lys48 ubiquitin chains, 250 nM E1, 8 μM E2-25K, 400 μM Ub (Sigma #U6253), and 100 μM Ub-sorHis were incubated in 2 mL of ubiquitination buffer (50 mM HEPES-NaOH [pH 7.5], 2 mM ATP, 5 mM $MgCl_2$, and 1 mM dithiothreitol [DTT]) at 37 ℃ for 2 h. Similarly, to synthesize Lys63 chains, 250 nM E1, 8 μM Ubc13, 8 μM MMS2, 400 μM Ub, and 100 μM Ub-sorHis were incubated in 2 mL of ubiquitination buffer at 37 ^{O}C for 2 h. Subsequently, ubiquitin chains containing Ub-sorHis at the proximal position were selectively purified using TALON resin and subjected to biotinylation in 2 mL of sortagging buffer (50 mM Tris-HCl [pH 7.5], 150 mM NaCl, 5 mM $CaCl_2$, and 12 μM Sortase) containing 12 mM GGGK-biotin at 37 ℃ for 4 h, followed by removal of unreacted Ub-sorHis using TALON resin. Ubiquitin chains were further purified by an ÄKTA protein purification system with a mono S cation exchange column (GE Healthcare) equilibrated with 50 mM ammonium acetate (pH 4.5) and eluted using a step gradient of NaCl (300–450 mM for Lys48 chain or 100–400 mM for Lys63 chain in 50 mM ammonium acetate [pH 4.5]).

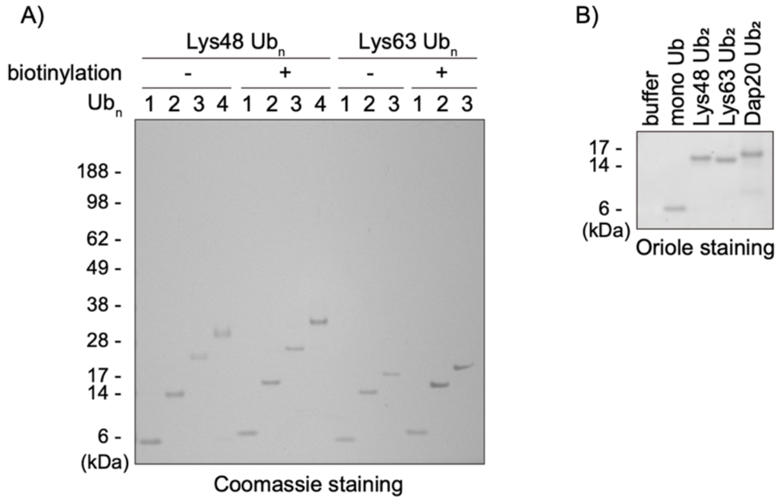

Figure S15: A) Lys48 and Lys63 chains with or without biotinylation purified through a cation exchange column. Purity of each fraction was assessed by SDS-PAGE and Coomassie staining. B) A set of biotinylated ubiquitin was analyzed by SDS-PAGE and oriole staining.

SDS-PAGE and Western blotting: Proteins denatured in 1× NuPAGE LDS sample buffer (Thermo Fisher Scientific) were separated by SDS-PAGE on 4–12% NuPAGE Bis-Tris gels (Invitrogen) with MES buffer. The gels were stained with oriole fluorescent gel stain (Bio-Rad) or Bio-Safe Coomassie Stain (Bio-Rad) or transferred to PVDF membrane (GE Healthcare). Western blotting was performed with HRP-conjugated streptavidin (200 ng/mL, Invitrogen #S911), mouse monoclonal antibody against GST (1:2,000, Santa Cruz #sc-138), and HRP-conjugated goat anti-mouse IgG (1:20,000, Promega #W402B) and visualized on a Fusion-FX7 EDGE (Vilber Bio Imaging) using ECL Prime Western Blotting Detection Reagent (GE Healthcare).

In vitro pulldown assays: To prepare whole cell extracts, HCT116 cells were washed with PBS and sonicated in lysis buffer (0.2% NP-40, 50 mM HEPES-NaOH [pH 7.5], 100 mM NaCl, and 10% glycerol) containing 10 mM iodoacetamide (IAA) and 1× complete protease inhibitor cocktail (Roche). Lysates were clarified by centrifugation (20,000 × g, 10 min, 4 ℃), and the protein concentration was determined by the BCA assay (Thermo Fisher Scientific). To immobilize ubiquitin on magnetic beads, 2 µg of each biotinylated ubiquitin was incubated with 40 µL slurry of Dynabeads M-270 Streptavidin (Invitrogen) in 500 µl of binding buffer (0.1% Triton X-100, 50 mM HEPES-NaOH [pH 7.5], 100 mM NaCl, and 10% glycerol) at 4 ℃ for 1 h and washed twice with binding buffer. For interactome analysis, the immobilized ubiquitins were incubated with 500 µg of whole cell extracts in 500 µL of lysis buffer at 4 ℃ for 1 h, washed four times with lysis buffer, and heated in 60 µL of 1× NuPAGE LDS sample buffer containing 5% β-mercaptoethanol at 70 ℃ for 10 min. For pulldown assays using recombinant proteins, the magnetic beads containing 100 ng of biotinylated ubiquitin were incubated with 1.5 µg of GST-tagged proteins in 500 µL of lysis buffer at 4 ℃ for 1 h, washed thrice with lysis buffer, and heated in 15 µL of 1× NuPAGE LDS sample buffer containing 5% β-mercaptoethanol.

Mass spectrometry-based interactome analysis:
On-beads tryptic digestion was performed using a KingFisher APEX (Thermo Fisher Scientific) as described previously with minor modifications.[6] Briefly, 1 µL of eluted proteins were reduced in 10 mM dithiothreitol (DTT) for 10 min at 70 ℃ and then alkylated with 15 mM iodoacetamide (IAA) for 15 min at room temperature. Based on a single-pot solid-phase-enhanced sample preparation (SP3) method,[7] alkylated proteins were denatured in 200 µL of 50% EtOH and conjugated to 40 µg of an equal mixture of hydrophobic and hydrophilic SeraMag SpeedBead carboxylate-modified magnetic particles (Cytiva), followed by desalting thrice with 400 µL of 80% EtOH. The magnetic beads were subjected to enzymatic digestion in 100 µL of 5 ng/µL Trypsin Gold (Promega) in 50 mM triethylammonium bicarbonate (TEAB) for 3.5 h at 37 ℃ and rinsed out with 100 µL of pure water. Digested peptides were acidified with a final concentration of 0.1% TFA and loaded into Evotip Pure (Evosep) according to the manufacturer's instruction. Desalted tryptic digests were analyzed by Evosep One (Evosep) coupled to an Orbitrap Fusion Lumos mass spectrometer (Thermo Fisher Scientific). Peptides were separated on a C18 analytical column (Aurora ELITE [1.7 µm × 75-

μm × 15-cm]; IonOpticks) using the defined Zoom 40SPD program. The Orbitrap Fusion Lumos mass spectrometer was operated in the data-dependent MS/MS mode with a mass range of 350–1300 m/z and resolution of 120,000 using Xcalibur software (Thermo Fisher Scientific). The most intense ions (cycle time 3 s) were selected for MS/MS fragmentation with the HCD of 30, isolation window at 1.6 m/z and maximum injection time at 22 ms in the centroid mode. The MS/MS spectra were searched against a UniProt knowledgebase (Homo sapiens sp_canonical (TaxID=9609), version 2024-10-02) using the Sequest HT search engine in the Proteome Discoverer software (version 3.2; Thermo Fisher Scientific). The precursor and fragment mass tolerances were set to 10 ppm and 0.02 Da, respectively. Methionine oxidation, protein amino-terminal acetylation, diglycyl-Lys, and Gln/Asn deamidation were set as variable modifications, and Cys carbamidomethylation modification was set as a static modification for database searching. Peptide identification was filtered at a 1% false discovery rate. Relative abundances were calculated by normalizing the label-free quantification values for each protein to a mean of 100. Principal component analysis (PCA) was performed using the Proteome Discoverer 3.2. Gene ontology analysis was performed using DAVID.[8,9]

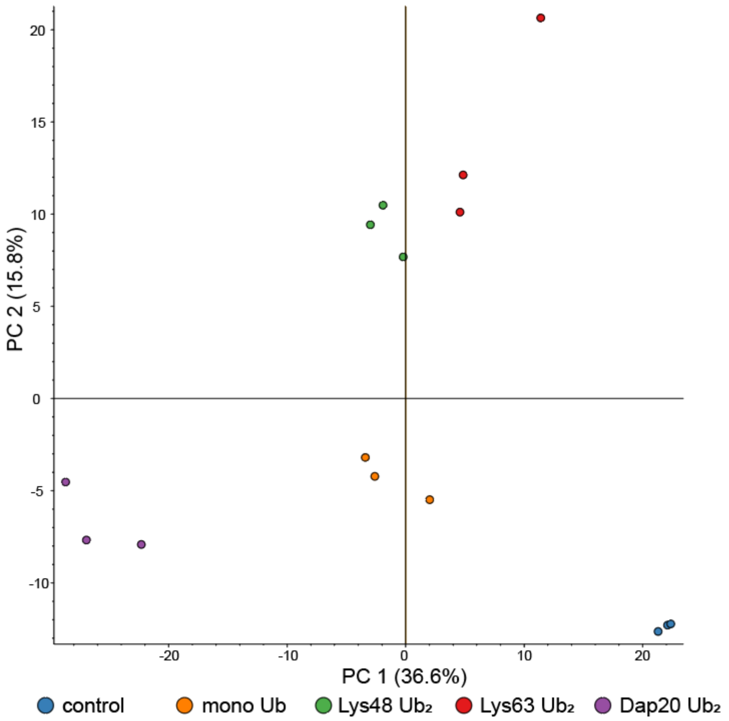

Figure S16: PCA of the mass spectrometry-based interactome analysis.

Table S1: The whole list of identifid proteins in the the mass spectrometry-based interactome analysis. The proteomics datasets have been deposited to PRIDE with the accession code PXD069069.

Deubiquitinating assay: 500 ng each of Dap20 diUb, USP2cc, and GST-USP39 were incubated in 20 μL reaction buffer (50 mM HEPES-NaOH [pH 7.5], 100 mM NaCl, 1 mM DTT, and 10% glycerol) at 37 ℃ for 16 h. The reaction was terminated by adding 10 μL 3× NuPAGE LDS sample buffer and heated at 70 ℃ for 10 min.

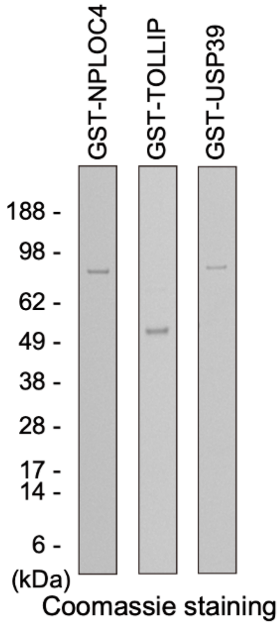

Figure S17: Purified recombinant proteins (GST-NPLOC4, GST-TOLLIP, and GST-USP39) were analyzed by SDS-PAGE and Coomassie staining.

[1] L. Chen, J. Cohen, X. Song, A. Zhao, Z. Ye, C. J. Feulner, P. Doonan, W. Somers, L. Lin, P. R. Chen, *Sci. Rep.* **2016**, *6*, DOI 10.1038/SREP31899.

[2] H. J. Jeong, G. C. Abhiraman, C. M. Story, J. R. Ingram, S. K. Dougan, *PLOS ONE* **2017**, *12*, DOI 10.1371/journal.pone.0189068.

[3] J. Shi, L. Kundrat, N. Pishesha, A. Bilate, C. Theile, T. Maruyama, S. K. Dougan, H. L. Ploegh, H. F. Lodish, *Proc. Nat. Acad. Sci U.S.A.* **2014**, *111*, 10131–10136.

[4] H. Tsuchiya, D. Burana, F. Ohtake, N. Arai, A. Kaiho, M. Komada, K. Tanaka, Y. Saeki, *Nat. Commun.* **2018**, *9*, DOI 10.1038/s41467-018-02869-x.

[5] Y. Sato, H. Tsuchiya, A. Yamagata, K. Okatsu, K. Tanaka, Y. Saeki, S. Fukai, *Nat. commun.* **2019**, *10*, DOI 10.1038/s41467-019-13697-y.

[6] Y. Iwasa, S. Miyata, T. Tomita, N, Yokota, M, Miyauchi, R. Mori, S. Matsushita, R. Suzuki, Y, Saeki, H. Kawahara, *J. Cell Biol.* **2025**, *224*, e202109010

[7] T. Müller, M. Kalxdorf, R. Longuespée, D. N. Kazdal, A. Stemzinger, J. Krijgsveld, *Mol. Syst. Biol.* **2020**, *16*, e9111.

[8] D. W. Huang, B. T. Sherman, R. A. Lempicki, *Nat. Protoc*. **2009**, *4*, 44.

[9] B. T. Sherman, M. Hao, J. Qiu, X. Jiao, M. W. Baseler, H. C. Lane, T. Imamichi, W. Chang, *Nucleic Acids Res.* **2022**, *50*, W216–W221.
